# Plant Essential Oils as Healthy Functional Ingredients of Nutraceuticals and Diet Supplements: A Review

**DOI:** 10.3390/molecules28020901

**Published:** 2023-01-16

**Authors:** Riccardo Matera, Elena Lucchi, Luca Valgimigli

**Affiliations:** 1 BeC s.r.l., Research & Development, Via C. Monteverdi 49, 47122 Forlì, Italy; 2 Department of Chemistry “Ciamician”, University of Bologna, Via S. Giacomo 11, 40126 Bologna, Italy

**Keywords:** essential oils, health, diet supplements, nutraceutical, functional food, antioxidant

## Abstract

Essential oils (EOs) are mixtures of volatile molecules endowed with health-promoting biological activities that go beyond their role as aromas and natural preservatives and can be exploited to develop functional foods and diet supplements. Their composition is briefly addressed along with regulatory aspects. The potential health benefit of human diet supplementation with EOs is outlined through a review of the recent literature on available clinical trials and preclinical research concerning EOs activity towards: (1) irritable bowel syndrome; (2) inflammatory bowel disease; (3) regulation of microbiota; (4) gastroprotection; (5) hepatoprotection; (6) protection of the urinary tract and diuresis; (7) management of metabolic disorders including hyperglycemia and hyperlipidemia; (8) anti-inflammatory and pain control; (9) immunomodulation and protection from influenza; and (10) neuroprotection and modulation of mood and cognitive performance. The emerging potential in such activities of selected EOs is given focus, particularly green and black cumin, bergamot, orange, myrtle, peppermint, sage, eucalyptus, lavender, thyme, lemon balm, ginger, and garlic.

## 1. Introduction

Essential oils (EOs) are mixtures of volatile lipid-soluble organic molecules traditionally obtained by steam distillation of fruits, flowers, leaves, stems, seeds, aromatic woods, and roots, typically representing the scent of the plant from which they are obtained. Other extraction methods such as dry distillation, mechanical pressing, solvent extraction, supercritical fluid extraction, and other high-efficiency extraction techniques may also be used, offering advantages for specific botanical sources [1,2]. Beside the distinctive scent, which grants their use in perfumery, EOs have a wealth of biological activities [3,4] that represent the basis of their ancient use in folk and traditional medicines [5]. They are also the heart of aromatherapy, a form of complementary alternative medicine that was born at the beginning of the 20th century as a result of the pioneering observations of French chemist René Maurice Gattefossé (1881–1950) and was much developed and expanded by Jean Valnet (1920–1995) [6] and by others [7,8,9].

Among the established uses of essential oils, the use in food products certainly stands out. Essential oils are the active parts of spices and herbs used since ancient times to flavor foods and aid their preservation; therefore, it is not surprising that they may continue to serve the same purpose in the modern food industry. Approximately 3000 EOs are currently known [10], and about 10% are used or can be used in food products. The U.S. Food and Drug Administration (FDA) lists about 300 essential oils and oleoresins among food additives that are classified as GRAS (Generally Regarded as Safe) Under Title 21 [11,12].

Most of their use is as aroma and flavor components in food or beverages; however, some more specific functions have been extensively investigated in recent years [5], particularly their role as natural food preservatives [13,14,15,16] owing to their antimicrobial properties [17,18] and to their antioxidant properties [19,20,21] or to an advantageous combination of both.

While a number of recent reviews has been dedicated to the use and potential of essential oils as food preservatives [13,14,15,16], comparably lower evidence has been given to other functional roles of essential oils or their components in food products, which are nonetheless accumulating a body of scientific support and substantial public interest: their health-promoting role beyond preservation and basic nutrition. Several functional roles of EOs have been extensively investigated and commercially explored in production animals’ nutrition, e.g., as additives to promote animal health and welfare and their action as immunomodulators, digestive stimulants, and natural substances that can increase the performance of animal products, replacing the need for antibiotics or other restricted additives [22,23]. Aim of this review is to offer an updated overview on the potential and on some viable applications of essential oils and their components as health-promoting ingredients of nutraceuticals and diet supplements or food products in human nutrition. Priority is given to the most recent advancements (last 10 years) without any aim to systematically cover the matter but rather to offer a picture of some promising directions for further development. Specific topics, such as the antimicrobial and antioxidant activities of essential oils, are deliberately excluded, as they have already been addressed by numerous recent reviews.

## 2. Composition of Essential Oils

Essential oils are—sometimes complex—mixtures of volatile apolar or moderately polar molecules comprising heterogenous structures that can, nonetheless, be clustered in classes of components bearing internal structural similarity and a common biosynthetic route. Many such components are hydrocarbons; i.e., they contain only C and H elements, while others also contain O (often referred to as oxygenated compounds) and more rarely N or S. Classification on the basis of the elemental composition is, however, less useful than following their biosynthetic route that dictates the overall structure. Along this line, most of the typical EO components belong to one of the two main families: *terpenoids* and *phenylpropanoids* [5].

**Figure 1:**
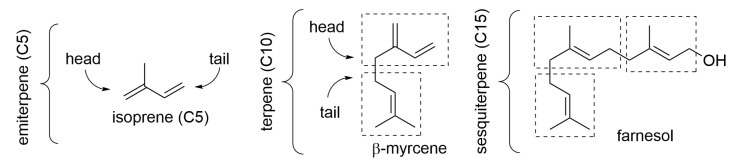
Structural features of volatile terpenes and terpenoids in essential oils.

**Figure 2:**
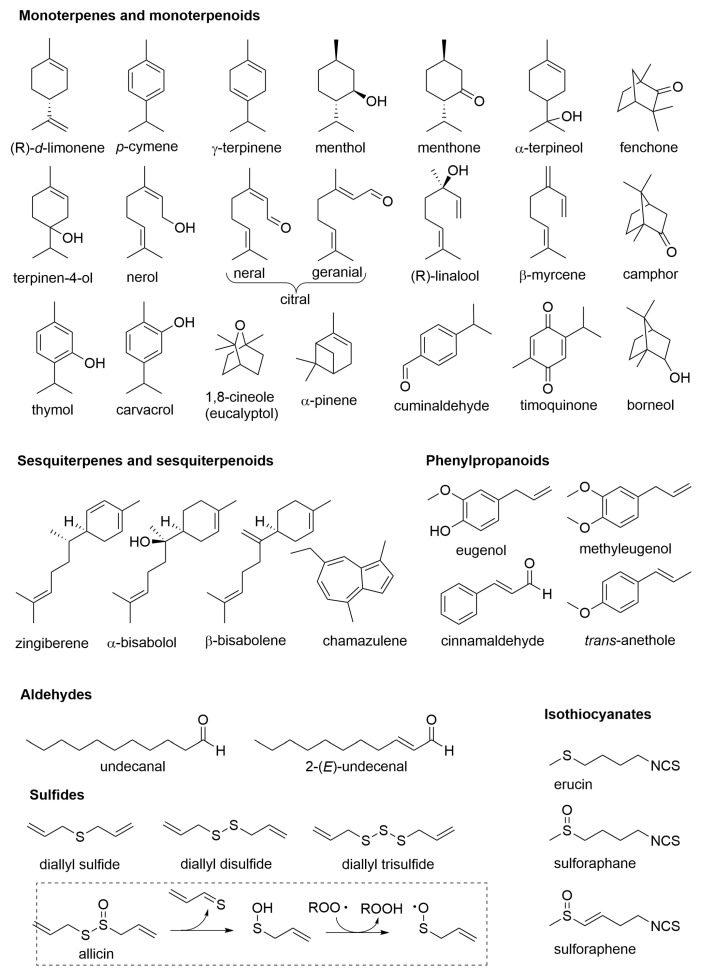
Examples of typical EO components classified by the structural family.

Terpenoids (Figure 1) are biosynthesized though the mevalonate pathway and are built by formally connecting isoprene units as C5 building blocks. (The actual building blocks are isopentenyl pyrophosphate and dimethylallyl pyrophosphate.) The majority of compounds contain two or three isoprene units and are called terpenes or sesquiterpenes, respectively. They owe their name to the wrong initial identification of the C10 unit as the recurrent building block, which was called terpene; therefore, the C15 molecules were identified as containing one and a half terpenes (from the Latin “semis” = half + “atque” = and, contracted to “sesqui”) (Figure 1) [5]. Although the plant contains components up to C30 (6 isoprene units), those larger than C15 (i.e., diterpenes and triterpenes) are rarely present in the EO since they are not sufficiently volatile to pass into the distillate. Formally, the term “terpene” refers only to hydrocarbons, i.e., those components having no heteroatoms, while the term “terpenoids” broadens to include oxygenated or heteroatoms-containing compounds although in common practice, this distinction is normally overlooked. A selection of common terpenoid components found in EOs is shown in Figure 2.

The second main family, the phenylpropanoids, comes from the shikimate biosynthetic pathway and comprises aromatic compounds substituted with an unbranched C3-chain on the ring. The side chain can be unsaturated and even truncated to a C2 or C1 by subsequent biosynthetic modification, and other functional groups can be present in the chain or in the ring [5]. Some examples of common phenylpropanoids are given in Figure 2. Along with terpenoids and phenylpropanoids, EOs may contain non-terpenic medium-chain hydrocarbons and aldehydes and simple alcohols and ethers.

*Allium* species yield EOs with a substantially different composition, consisting mainly of sulphurated volatiles. Fresh garlic (*Allium sativum* L.) EO, for instance, contains mainly sulfides, disulfides, and trisulfides along with highly unstable thiosulfinates such as allicin (Figure 2). Allicin, however, is not present in garlic clove but formed upon mechanical disruption of the tissues and transformation of non-proteinogenic amino acid precursor alliine (S-allyl cysteine) by released enzyme alliinase [5]. The EOs from *Brassicaceae*, such as mustard (*Brassica nigra*), salad rocket (*Eruca* spp.), broccoli (*Brassica oleracea* var. *Italica*), and daikon (*Raphanus sativus*), are instead characterized by volatile isothiocyanates containing both S and N and providing the distinctive spicy-pungent flavor along with the bioactivity (Figure 2) [24,25,26,27].

Different botanical species offer different EOs with a characteristic composition, which is, however, subjected to (sometimes large) variability associated with geographical origin, soil composition, climate, cultivation and harvesting methods and time, EO extraction method, etc. This clearly results in different properties of the EOs. To partly account for such diversity, the concept of *chemotype* was introduced; i.e., EOs from different plants of the same botanical species can be sub-classified according to the main components or to the relative abundance of main and/or characteristic components [5].

A chemotype (CT) describes the subspecies of a plant that have the same morphological characteristics but produce different quantities of chemical components in their EOs. This is particularly important in the family of *Labiatae* or *Lamiaceae*, comprising lavender, lemon balm, peppermint, basil, rosemary, sage, thyme, and others. Some examples are illustrated in Table S1 in the Supplementary Materials.

## 3. Nutraceuticals, Diet Supplements, and Functional Food

A well-established definition of *functional food* is that given by the International Food Information Council (IFIC) as a “*food or dietary components that may provide a health benefit beyond basic nutrition*” [28]. It is interesting to compare it with the definition of *nutraceutical*, whose name comes from the crasis of the terms “nutrition” and “pharmaceutical”, given by the Foundation for Innovation in Medicine as “*any substance that can be considered a food or part of a food that provides medical or health benefits, including the prevention and treatment of disease. Such products may range from isolated nutrients, dietary supplements and diets to genetically engineered “designer” foods, herbal products, and processed foods such as cereals, soups, and beverages*” [28]. It may appear that the two definitions are overlapping, at least in some respect, or perhaps that nutraceuticals include functional food as a possible case. 

A sharper definition of functional food is that given by the European Food Safety Authority (EFSA) as “a food, which beneficially affects one or more target functions in the body, beyond adequate nutritional effects, in a way that is relevant to either an improved state of health and well-being and/or reduction of risk of disease. A functional food can be a natural food or a food to which a component has been added or removed by technological or biotechnological means, and it must demonstrate their effects in amounts that can normally be expected to be consumed in the diet” [29]. This last definition suggests a distinction between food products with a healthy attribute (functional food) and dietary products formulated in the form of a pill or tablet or capsule, i.e., in a pharmaceutical-like form. This last type of dietary products might instead be identified with diet supplements which, according to the EFSA, are “concentrated sources of nutrients (i.e., mineral and vitamins) or other substances with a nutritional or physiological effect that are marketed in ‘dose’ form (e.g., pills, tablets, capsules, liquids in measured doses). A wide range of nutrients and other ingredients might be present in food supplements, including, but not limited to, vitamins, minerals, amino acids, essential fatty acids, fibre and various plants and herbal extracts” [30].

It should be noted instead that a legal definition of nutraceutical is missing, and in different countries, different local regulations might classify such products differently. Although a healthy function is the aim, a clear distinction needs to be maintained between food products (in general) and pharmaceutical products; therefore, health claims that are admissible for healthy food and diet supplements are strictly regulated in the EU, as in USA and in other countries [31].

With this in mind, it is apparent that the biological efficacy and health benefit offered by nutraceuticals is sometimes comparable to that of drugs to the point that they can both help prevent disease and represent an alternative therapeutic approach, particularly for chronic conditions, and particularly when they arise from environmental factors or from diet. The main disturbances that EOs inserted in nutraceuticals could help handle are illustrated, following an order based on a bottom-up sequence along the digestive tract.

## 4. Essential Oils Helpful in the Irritable Bowel Syndrome (IBS)

Irritable bowel syndrome (IBS) is a complex gastrointestinal (GI) inflammatory condition associated with abdominal pain, diarrhea, or constipation that can change over time as well as flatulence, spasms, and other individually variable symptoms with still incompletely understood causes that likely include diet. Since its pharmacological management is often difficult, a dietary intervention including the use of diet supplements appears a valuable option [32].

Peppermint EO (*Mentha piperita.* L.) has perhaps the best-established efficacy against IBS symptoms. Its main components, i.e., menthol and menthone, have well-documented antispasmodic and muscle-relaxing activity [33]. Menthol, in particular, was shown to block Ca^2+^ channels in rats and guinea pig [34]; for this reason, both menthol and peppermint EOs are effective in decreasing motor activity in GI smooth muscle and in providing relief for colon spasms, with proven beneficial activity during colonoscopic examination [33]. In addition, peppermint EO contains 1,8-cineol with proven anti-inflammatory activity [33]. In several randomized, double-blind, placebo-controlled clinical studies, peppermint EO showed significant efficacy in alleviating the severity of abdominal pain and in reducing abdominal distension, stool frequency, and flatulence. Improvements were significantly better than after placebo, with no significant incidence of side effects [32,35,36]. The effects of peppermint EO have been attributed to its antimicrobial and antispasmodic activity [32]. A recent systematic review and meta-analysis on the efficacy of peppermint EO in improving IBS analyzed, overall, 10 placebo-controlled randomized trials with minimum duration of four weeks, involving a total of 1030 patients [37]. Both small-intestine-release microencapsulated oil (182–540 mg/day) and regular peppermint oils (2 mL/day) were investigated in the different trials. The analysis demonstrated that peppermint EO was more effective than placebo in all 10 trials in terms of either global symptoms or abdominal pain, concluding that peppermint is an efficacious treatment for IBS [37].

Anise EO (*Pimpinella anisum* L.), obtained by hydrodistillation from dry ripe fruits and containing *trans*-anethol as the main component (93.3%), formulated (200 mg) in enteric-coated caps with vegetable oils and beeswax (AnisEncap) was evaluated against Colpemin^®^ (peppermint 0.2 mL, 187 mg) in a three-armed, double-blind clinical study on 120 IBS patients who received three capsules/day for 4 weeks plus 2 weeks of follow-up. At the end of the trial, 75% of patients who received anise EO were free from IBS symptoms vs. 35% in placebo and 52.5% in Colpemin^®^ groups. Overall, anise EO showed higher efficacy and fewer side effects than peppermint EO and was judged a promising, well-tolerated therapy for IBS symptoms [38].

Garlic (*Allium sativum* L.) has documented beneficial effects toward gastrointestinal symptoms such as colic pain, flatulent colic, and dyspepsia. The EO is characterized by prevailing sulphurated compounds (84–99%) with diallyl trisulfide (37–46%), diallyl disulfide (18–36%), and methyl allyl trisulfide (8–10%) as the major components [39]. All of them are formed from the thiosulfinate allicin, which is unstable and, in turn, is formed on chopping or chewing cloves, i.e., upon mechanical disruption of the vegetal tissues, which starts the alliinase-catalyzed hydrolysis of cysteine-derived alliin [40]. Allicin spontaneously decomposes, releasing allylsulfenic acid, which, along with other sulfenic and selenenic acids, is one of the most potent peroxyl-radical-trapping antioxidants known in nature [40,41,42]. Besides the excellent antioxidant activity, garlic EO components are known to have excellent antibacterial activity, and it is likely the combination of these two properties that mostly contributes to IBS symptoms improvement [43].

Thymol has well-known beneficial activity in gastrointestinal symptoms, with mechanisms that are still matter of investigation besides the established antioxidant [19] and anti-inflammatory activity [43]. A recent study demonstrated that thymol (50 mg/kg b.w.) counteracted IBS induced by physical stressors in the rat model [44]: it normalized gut transit time and abdominal withdrawal reflex, and it decreased intestinal hypermotility. The study concluded that oral thymol administration could be a potential option to handle leaky gut with subsequent soothing of the IBS symptoms possibly by regulating the serotonin receptor (5-HT_3_AR) in the intestinal epithelium, as confirmed by molecular docking analysis [44]. Another recently identified mechanism for thymol’s anti-inflammatory activity and its modulation of gut motility relies on its interaction with the gastrointestinal endocannabinoid system [45]. Thymol modulates the expression of the CB1, CB2, TRPV1, and OR1G1 mRNAs in the duodenum and ileum as well as the mRNA levels of enzymes involved in the biosynthesis and degradation of endocannabinoid molecules. Therefore, the effects of thymol in promoting gut health are also potentially mediated by the activation of these receptors [45].

The essential oil of *Zataria multiflora* Boiss, a thyme-like plant belonging to the *Lamiaceae* family that grows wild in Iran, having carvacrol (12–71%) and thymol (15–49%) as the main components, was reported to relieve symptoms of IBS without any significant adverse effect when administered to volunteers at a daily dose of 60 drops of a 2% solution. The efficacy was attributed to the anti-inflammatory and anti-spasm activity of thymol and carvacrol [46]. A recent randomized clinical trial involving 150 volunteers evaluated a combined administration of *Zataria multiflora* EO (250 mg/day) with *Trachyspermum copticum* L. EO (500 mg/day), a plant used in Persian medicine as anti-microbial and anti-inflammatory (ZT capsules), against placebo and against 135 mg of mebeverine as positive control. After the fourth week of intervention, symptoms of pain, bloating, and reflux showed a significant decrease in the ZT group compared to both the placebo and mebeverine groups (*p* < 0.05), which confirmed the positive effect of ZT on IBS symptoms. The efficacy was mostly attributed to thymol and carvacrol [47]. A diet supplement (Enterofytol^®^) containing a combination of fennel (*Foeniculum vulgare* L.) essential oil (25 mg) and turmeric (*Curcuma longa* L.) extract (42 mg of curcumin) was evaluated during 60 days in two clinical studies: a double-blind, placebo-controlled trial on 120 IBS patients and a “real-life” study on 211 IBS subjects from two countries (Italy and Belgium). It was proven to significantly improve all IBS symptoms and the quality of life of subjects irrespective of age, sex, and initial severity of IBS symptoms [48].

Recent studies highlight the association of IBS symptoms with the relative abundance of some species in intestinal microbiota [49]; for instance, there was found inverse correlation between the abundance of *Akkermansia muciniphila* and pain reduction in IBS patients [50]. In addition, the relative abundance of fungal strains, such as a decrease in *S. cerevisiae* and an increase *C. albicans* abundance, correlates with the decrease of anti-inflammatory cytokines (e.g., IL-10) and the increase in pro-inflammatory mediators (IL-6m IL-8, TNF-α), thereby suggesting that modulation of yeast abundance with selectively antimicrobial essential oils could ameliorate IBS symptoms [49]. Among the tested EOs, a combination of bitter orange EO (*Citrus aurantium* var. *amara*) showed the highest selectivity in inhibiting IBS-isolated strains such as *C. albicans* while saving beneficial strains such as *S. cerevisiae* or commercial probiotic strains such as *L. acidophilus*, *L. casei*, and *S. boulardii*, with maximum selectivity when dosed < 2% [49]. The study concluded that combination of 1% bitter orange EO dispersed in *Vitis vinifera* hydrolate, which also contained 1% terpenic components (mainly limonene, linalool, and cis-geraniol) and was shown to possess immunomodulatory activity, would represent an optimized oral supplement for IBS patients [49].

## 5. Essential Oils Helpful in Inflammatory Bowel Disease (IBD) and in the Prevention of Colorectal Cancer (CRC)

Inflammatory bowel disease (IBD) is a chronic inflammatory disease of the gastrointestinal tract that comprises two phenotypical conditions, namely ulcerative colitis (UC) and Crohn’s disease (CD). It affects approximately 1.6 million individuals in USA (0.5% of population, with a growth of 70,000 new cases every year) and 0.2% of the European population [51], with a growing number of cases in South America, the Middle East, and Asia. IBD is distinct from IBS, and it is characterized by GI symptoms such as rectal bleeding and diarrhea, bloating, abdominal cramping, pain, reduced appetite, unintended weight loss, and fatigue. Although causes are incompletely understood, IBD is normally associated with an over-reactive immune system and is related to genetic and environmental factors. IBD is also associated with a 30-fold higher risk of developing colorectal cancer (CRC) owing to the massive proliferation required to repair the intestinal tissue injury [52]. Diet and diet supplements are actively investigated as complementary therapeutic approaches to reduce the need for pharmacotherapy or surgery. Several dietary medicinal plants and essential oils have shown promising results; however, no dietary intervention has been clinically validated yet. Animal studies can offer a meaningful insight using well-established translational models in rats or mice, such as treatment with acetic acid (AA) or dextran sulfate sodium (DSS) or oxazolone (OZ) to induce ulcerative colitis (UC) or with trinitrobenzenesulfonic acid (TNBS) to model Crohn’s disease (CD) [52].

A recent review analyzed the intestinal anti-inflammatory and antioxidant activity (modulation of markers TNF-α, IL-1, IL-7, IL-10, IL-11, IL-12, and mRNA of NF-kβ, MPO, COX-2, and PPARγ) of several essential oils orally administered in the rat translational model for ulcerative colitis (UC) or for Crohn’s disease (CD), finding that *Zanthoxylum bungeanum* (rich in terpinen-4-ol, eucalyptol, xanthoxylin); *Zanthoxylum myriacanthum* (limonene, α- and β-phellandrene, α-pinene, o-cymene); and *Curcuma longa* (curcumin) where effective on UC at doses in the range 20–80 mg/Kg b.w, while *Foeniculum vulgare* (trans-anethole, fenchone, methyl chavicol, limonene); *Zingiber officinale* (zingiberene, α-curcumene, β-bisabolene, α-sesquiphellandrene); *Ocimum basilicum* (linalool, β-pinene, trans-verbenol, α-terpinolene); and *Cymbopogon martini* (geraniol) required a minimal dose of 200, 100, 160, and 120 mg/Kg b.w, respectively [52]. 

A spearmint (*Mentha spicata* L.) hydroalcoholic extract was effective in reducing IBD colon lesions, diarrhea, and inflammation in the rat model, with reduction of iNOS expression and histological markers, which was attributed mainly to the antioxidant and anti-inflammatory properties of the extract [53].

Owing to the combination of antioxidant and anti-inflammatory activities, thymol and thyme (*Thymus vulgaris* L.) EO can help treat UC and CD, as shown with young pigs, by oral administration of thymol (50 mg/kg) [54]. Both thymol and carvacrol share antioxidant properties and potent anti-inflammatory activity [55], which justify the good protective effect toward TNBS intestine lesions by thyme (*Thymus vulgaris* L.) and oregano (*Origanum onites* L.) EOs having both thymol and carvacrol as main components comprising up to 70% of the oil [56].

Coriander (*Coriandrum sativum* L.) belongs to the *Apiaceae* family and is a widely used dietary spice as a part of curry powder. It has long-standing medicinal use, with properties largely attributed to the essential oil, which can be extracted by steam distillation both from the fruits and from the leaves, with substantially different composition that also greatly varies with the geographical origin and ripening degree. The main components of immature fruits are often geranyl acetate (46%), linalool (11%), nerol (1–2%), and neral (1–2%). On ripening, fruits EO become progressively richer in linalool (70 to 88%), while geranyl acetate becomes less prominent (2–4% in the middle stage), and cis-dihydocarone (2–4%) becomes characteristic. Leaves’ EO contains linalool as the main component (65–75%), camphor (3–7%), γ-terpinene (1–14%), α-pinene (1–3%), and β-pinene (1–2%) and variable amounts of medium-long chain aldehydes such as decanal, undecanal, dodecanal, 2(*E*)-undecenal, 2(*E*)-dodecenal, etc. [57]. Coriander has been traditionally used in Ayurveda medicine for gastrointestinal disorders, diarrhea, vomit, spasm (fruits), anxiety, insomnia, depression, headache, and skin disease (leaves) [57]. Its components (both in leaves and fruits) support marked anti-inflammatory activity; for instance, fruit EO counteracted carrageenan-induced paw edema in rats at 50–200 mg/Kg, with the highest dose matching the efficacy of diclofenac 5 at mg/kg [58]. When it was evaluated in an experimental model of UC (AA-induced in rats), coriander EO per os at 0.5 mL/kg b.w. showed reduction of colon swelling compared to control group and significant reduction of MPO levels, ulcer severity, and total colitis index, promising potential for the amelioration of IBD in humans [59].

Citrus fruits and their EOs have good potential in mitigating IBD symptoms due to their anti-inflammatory activity associated with regulation of gut microbiota, which has recently been reviewed [60]. Among them, lemon (*Citrus limon* L.) EO has traditional use in aromatherapy to relieve fatigue [9], which is one of the IBD symptoms. It is also among the most abundant natural sources of limonene (up to 85%), which was recently shown to be a potent protective agent in a translational UC model [61]. The EOs of *Agastache mexicana* ssp. *mexicana* and ssp. *xolocotziana* and their components limonene (ca. 10% of the two oils [62]) and ulegone (ca. 80% in spp. *xolocotziana* only [62]) were tested for antinoceptive activity in an abdominal pain model, where limonene showed the highest antinociceptive efficacy that was dose-dependent in the range 3–100 mg/Kg b.w., and it also significantly decreased hyperalgesia, pathological biomarkers, and colonic inflammatory cytokines in the oxazolone-induced colitis model. Histological analysis showed that 177.8 mg/Kg b.w. limonene gave protection equivalent to 100 mg/Kg b.w. of the reference drug sulfasalazine according to the disease activity index, while equivalent protection to 100 mg/Kg b.w. sulfasalazine was reached with 30 mg/Kg b.w. limonene concerning the colon damage index [61].

A few studies suggest that lavender EO (*Lavandula officinalis* L.) has good potential in treating UC, partly owing to its probiotic activity [63], although the actual mechanism and contribution of individual components has not been clarified. A very recent study [64] based on the network pharmacology approach analyzed the network contribution of individual EO components in (DSS)-induced UC in mice, identifying the regulation Th17 cell differentiation as the therapeutic target of lavender EO and the reduction of signaling factors EGFR, TNF-α, and IFN-γ as the key markers of its action. The study concluded that the EO has significant activity in reducing colonic mucosal damage and has great potential for the medical treatment of UC [64].

Arizona cypress (*Cupressus arizonica* Greene) EO obtained by hydro-distillation of the dried fruits (containing 72% α-pinene) was demonstrated to have therapeutic effect on AA-induced colitis in rats at a dose as low as 0.5 mg/Kg. b.w., reducing macroscopic lesions, necrosis, and inflammation and proving much more effective than the flavonoids-rich hydroalcoholic extract [65]. The efficacy was attributed to α-pinene, which has also shown anti-inflammatory and protective activity in other IBD translational models [66,67]; therefore, it is expected that other EOs rich in α-pinene would have similar potential.

Myrtle (*Myrtus communis* L.) has been used traditionally for the treatment of diarrhea, hemorrhoids, peptic ulcer, urethritis, inflammation, hemorrhagic ulcers, pulmonary, and skin diseases. The EO contains α-pinene as the main component, followed by limonene, 1,8-cineole, and linalool; therefore, it promises beneficial activity in IBD. This was confirmed in a recent study on AA-induced UC model in rats, where myrtle EO showed significant reduction of MPO and of the severity of lesions, ulcer area, and ulcer index at low doses (62.5 to 250 μL/Kg b.w.), while the efficacy faded away at higher doses [68]. At the dose of 125 mg/Kg b.w., the EO had efficacy comparable to 4 mg/Kg b.w. prednisolone or 100 mg/Kg b.w. mesalazine, which were used as positive control [66].

A nanoemulsion containing a combined herbal preparation composed of fennel, anise, chamomile, and pomegranate fruit peel in linseed oil was evaluated in a TNBS-induced Crohn’s model in rats; it showed improved performance over the single plant extracts and was judged a promising approach for IBD dietary treatment [69].

Colorectal cancer (CRC) is the second leading cause of cancer death in Europe, and it is associated with intestine microbiota and chronic inflammation such as IBD. Critical reviews and discussions on the EO components that could provide protection against CRC have recently become available [52]. In a very recent study aimed at identifying EO components effective in the prevention of CRC, six representative molecules with previously reported in vitro efficacy, namely monoterpenoids carvacrol, thymol, and geraniol; sesquiterpene β-caryophyllene; and phenylpropanoids cinnamaldehyde and eugenol, were comparatively tested on NCM-460, a normal human mucosal epithelial cell line; Caco-2, a human colon epithelial adenocarcinoma cell line; and SW-620, colon cancer cells derived from a lymph node metastatic site [70]. In the tested concentration range, carvacrol (150–1000 µM) and thymol (165–1650 µM) did not show selectivity toward cancer cells, while β-caryophyllene at the lower dose range (150–240 µM) seemed to promote Caco-2 proliferation, while at higher doses (360–480 µM), it was similarly toxic on all cell lines. Instead, both cinnamaldehyde at a concentration of 75 µM and eugenol at 800 µM, after 72 h of treatment, were capable of inducing apoptosis, necrosis, and a cell cycle slowdown in Caco-2 and in SW-620 but not in NCM-460 cells. Cinnamaldehyde had higher selectivity of SW-620, while eugenol for Caco-2 proved effective in both the prevention and treatment of CRC [70]; therefore, EOs rich in these compounds such, as clove bud and cinnamon leaves oils (up to 85% in eugenol) and cinnamon bark (up to 75% in cinnamaldehyde), deserve further attention [70].

## 6. Essential Oils in Probiotic Food and Supplements for Regulation of Gut Microflora

According to the World Health Organization, probiotics are living strains of microorganisms to be consumed in suitable amounts in order to provide health benefits to the host by modulating activity and composition of gut microbiota [71]. These types of microorganisms consist mainly of bacteria but also include yeasts, which are naturally present in fermented foods, may be added to other food products, and are available as dietary supplements. Probiotics act usually in the gastrointestinal tract, and their viability depends on the baseline microbiota, probiotic strain, and gastrointestinal tract region [72]. They may influence the intestinal microbiota during their temporary colonization in the gut mucosa so that they must be consumed nearly on a daily basis to provide a real positive impact [73]. For this reason, food represents a preferred way to administer probiotics, followed by enriched dietary supplements. Probiotics should not be confused with prebiotics, which are typically complex carbohydrates (such as inulin and other fructo-oligosaccharides) that microorganisms in the gastrointestinal tract use as metabolic fuel [74]. Commercial products containing both prebiotic sugars and probiotic organisms are often called “synbiotics”.

Some essential oils have greater minimum inhibitory concentration (MIC) values for probiotics compared to pathogens. Such a phenomenon makes it possible that both probiotics and essential oil are administered together to treat pathogenic infection in the human gut [75]. They can be combined to form essential oil-flavored fermented milk products, such as flavored curd beverages or flavored yogurt. Among fermented foods, yogurt represents a key example of source of probiotic microorganisms such as *Lactobacillus delbrueckii* subsp. *bulgaricus* and *Streptococcus thermophiles,* which should survive intestinal transit in order to be claimed as probiotic. Yogurt consumption promotes probiotic bacterial growth by changing the gastrointestinal tract bacteria and also supports immune system contributing to the overall health and preventing age-related weight gain [76].

The research on yogurt production has empowered technologies and processes, particularly when yogurt is fortified with different nutrients such as organic acids, microelements, fruit, vegetable extracts, phytochemicals, and essential oils. The combination should maintain pleasant sensorial and organoleptic properties along with meeting supplementary nutritional needs [77] so as to improve their marketability.

Different examples of EOs-fortified yogurt studied to extend the normal shelf life of products due to the inherent antibacterial, antifungal, and antioxidant properties of EOs, thereby exploiting their potential use as natural preservatives, have been extensively reviewed [15]. Research in biopolymer nanoemulsions loaded with EOs paved the way to achieve oxidative stability, thermostability, shelf life, and supplementary biological activity in food products, minimizing synthetic preservatives [78]. Examples include *M. officinalis* essential oil, which was incorporated in microcapsules, showing that the antioxidant activity of yoghurt samples was increased [79].

The main limitation for using EOs in the food industry is their organoleptic effect due their strong aroma [14] even though the change in organoleptic properties in yogurt may not necessarily be perceived as a drawback. Therefore, in designing EOs-fortified yogurt with the aim to convey functional properties to food, the contribution of the EO to the final flavor cannot be overlooked, and both aspects, namely functional properties and final flavor, need to be considered and optimized.

*Ferulago angulata* ethanolic extract and essential oil were added to probiotic yogurt (*Lactobacillus acidophilus* and *Bifidobacterium bifidum* bacteria), and their effects on the survival of probiotic bacteria during storage were investigated. The survivability of the both probiotic bacteria in *Ferulago angulata* EO (0.03%)-fortified yogurt was significantly higher than that of the control, resulting in good profiling in protein, physicochemical, microbial, and sensory tests [80]. Viability of the probiotics, antioxidant activity, and organoleptic acceptability of yogurt containing EOs of zataria, basil, or peppermint was investigated, aiming at a yogurt with improved antioxidant potential. Peppermint and basil samples showed both good antiradical activity and sensory acceptability [81]. Eucalyptus (*Eucalyptus camaldulensis*) and myrrh (*Commiphora myrrha*) EOs were investigated in fortified buffalo set yogurt to assess their benefits in terms of health functionality. The effects on sensory, texture, antibacterial activity, total phenol content, and antioxidant activity of yogurt were evaluated, and eucalyptus oil (0.9%)-fortified yogurt showed the highest antioxidant activity and total phenolic content. The results showed that both eucalyptus and myrrh oils can be applied to yogurt to improve its beneficial properties both in terms of physical characteristics and of beneficial impact on human health [82].

Other fermented foods that contain probiotic microorganisms include beverages such as fermented milk. In an independent study, three beverages were prepared with probiotic curd with varying concentration of essential oils of *Coleus aromaticus, Rama tulasi*, and *Shyama tulasi* [83]. These beverages were then grown with common enteric pathogens in equal concentration, measured by CFU count. The sample beverages were found to be highly effective in inhibiting the growth of the pathogen. The shelf life of the beverages was also found to be significantly higher than for normal probiotics. The test results can be interpreted as the beverage’s capacity for prevention of enteric pathogens [83]. The above examples clearly show that both probiotics and essential oils have a great potential in terms of their beneficial effect against microbial gut infection, and their combined use can afford even more beneficial functional food [75].

## 7. Essential Oils for Gastric Protection and to Alleviate Peptic Ulcer

Peptic ulcer is a chronic pathology affecting a relevant portion of the population worldwide (as much as 10% for at least some time). Based on the main localization of lesions, it is often classified into gastric ulcer or duodenal ulcer. It is a multifactor condition that can be triggered by several causes, including the use of anti-inflammatory drugs, abuse of alcohol, inappropriate diet, and emotional stress. There is strong association between gastric and duodenal ulcers and the infection by *Helicobacter pylori*, which causes inflammation of the mucosa [84]. Studies have shown that pro-inflammatory cytokines and oxidative homeostasis have a key influence in the onset of gastric ulcer, which sees an overboost of oxidative stress counterbalanced by activation of the nuclear factor-erythroid-2-related factor 2 (Nrf2) signaling pathway, which upregulates the biosynthesis of endogenous antioxidant enzymes [85]. Although several effective pharmaceutical treatments are available for peptic ulcer, its chronic and recurrent nature makes a dietary intervention aided by supplements a valuable alternative. Many essential oil components have shown efficacy in ameliorating peptic ulcer, and they have been reviewed [84]. Additionally, several studies on raw essential oils have recently become available although none has yet received clinical validation. 

*Juniperus communis* L. leaf extract and EO have anti-ulcer activity. In the animal models at doses of 50–100 mg/kg b.w., juniper demonstrated reduction of gastric ulceration caused by salicylic acid, serotonin, indomethacin, or alcohol, while in guinea pigs, it protected from histamine-induced duodenal lesions. Of interest, it did not alter gastric pH, total acidity, and peptic acidity in gastric juice [86]. In humans, juniper assists in the digestive function and protection of inflamed gums [87]. 

Thyme oil (*Thymus vulgaris* L.) constituents demonstrated beneficial effects in gastric ulcer animal models compared to controls. This holds particularly for carvacrol, which inhibited damage to the gastric epithelium when administered orally (12.5–50 mg/kg) to rats prior to induction of acute gastric lesions. The protection was evident also with different methods of gastric damage induction, and interestingly, it did not alter gastric juice volume and acidity [54]. Carvacrol also showed reduction of gastric lesion after their induction in 14-day treatment of rats in the dose range 25–100 mg/kg b.w. Further, α-terpineol was gastroprotective when dosed orally (10 to 50 mg/kg) prior to administration of ulcer-inducing agents, without changes in gastric acid secretion [54]. The protection is mainly attributed to inhibition of COX-2 and of TNF-α biosynthesis [54] although recent evidence obtained on structurally related thymol (co-principal component of thyme EO) possibly suggests its bioactivity could also be mediated by modulation of the endocannabinoid system [45].

An additional emerging mechanism explaining the ulcer protecting activity of *Thymus* species is their marked activity against *Helicobacter pillory*, particularly expressed by thymol and carvacrol but possibly enhanced by other components. This is supported by two very recent microbiological investigations on *T. vulgaris* EO and *T. capitatus* (= *Thymbra capitata* L.; Spanish oregano) and boosting thymol and carvacrol as characteristic components [88,89]. On comparing the EOs of three *T. capitatus* varieties from Cyprus, having 47–57% thymol, 5.7–8.5% carvacrol, 12–15% *p*-cymene, and 5–10% γ-terpinene along with >30 other components, MIC ranged 0.25–0.5 mg/L and MBC 0.5–1 mg/L, with the highest activity being recorded for the specimen having lower thymol and higher *p*-cymene and γ-terpinene. Besides the intriguing suggestion of a possible synergism among components, the activity against *H. pylori* was excellent for all the testes EOs, highlighting the potential in ulcer prevention [90]. Similarly, *T. vulgaris* EO was proven active against three strains of *H. pylori* isolated from gastric human biopsies in hospitalized cases of gastritis, ulcer, and malignant tumor. In all the three pathological cases, MIC and MBC were 62.5 and 125 mg/L, respectively [88].

The EO of *Thymus hirtus* ssp. *algeriensis* Boiss, a thyme species distributed in North Africa and locally used as culinary herb, was shown to express relevant healing of HCl/ethanol-induced ulcers in rats [89]. Oral administration at doses of 54, 117, and 180 mg/kg b.w. showed marked decrease in the number of ulcer lesions and of the ulcer index, accompanied by higher pH and mucus production. Biochemical analysis also revealed increased antioxidant defenses in the treated vs. control groups, as witnessed by increased levels of GSH, SOD, CAT, and GPx, accompanied by a decrease in TBARS. While these findings are somewhat different from those recorded with other *Thymus* species, they are explained by a markedly different composition of the EO, which contained linalool, 1,8-cyneol, camphor, and viridiflorol as the main components and conceivably acted with an anti-inflammatory [55] via the induction of antioxidant enzymes as well as by increased secretion of mucus, i.e., by the increasing of gastric mucosal defensive mechanisms [68]. 

*Pistacia atlantica* L., a species of pistachio tree known by the English common name Mt. Atlas mastic tree, offers an oleoresin that contains about 20–25% essential oil. The EO obtained by hydrodistillation of the resin contains predominantly α–pinene (93%) along with β-pinene (1.7%), limonene (0.6%), camphene (0.6%), and other minor components [91]. It was shown to inhibit six clinical strains of *H. pylori* isolated from human lesions with MIC in the range 0.28–1.1 mg/mL and to afford significant protection in ethanol-induced ulcers in rats. Administered *per os* 25–100 mg/kg b.w., it gave dose-dependent protection with a reduction of ulcer index at all doses and EC_50_ of 12.32 mg/Kg. At the dose of 100 mg/Kg, it significantly outperformed the reference drug ranitidine (used at 50 mg/Kg; *p* < 0.01), which performed similar to 25–50 mg/Kg of the EO. Interestingly, the EO showed no toxicity in rats up to a dose of 2000 mg/Kg b.w., and it can be considered a promising and safe natural remedy for gastric ulcer treatment [92].

*Citrus aurantium* L. (*Rutaceae* family) has widespread traditional use in treating gastritis and other gastric disorders; its essential oil, extracted from the outer peel by cold pressing, is commonly used as a flavoring agent. Fresh peel is also used to flavor drinks, while the dried peel is traditionally used in the preparation of astringent digestive teas to help treat gastritis. The efficacy of orange EO as a gastroprotective substance has been demonstrated in different studies [91,93,94]. Morales et al. reported gastroprotection in young rats treated with ethanol or indometacine to induce ulcers, with maximum efficacy at the dose of 250 mg/kg b.w. At this dose, both orange EO and pure limonene (the main component of the oil, tested at 245 mg/kg b.w) offered effective protection by increasing gastric mucus production sustained by the biosynthesis of PGE2 without altering H^+^ secretion or serum gastrin and maintaining the levels of GSH [91]. Subsequent studies on a similar model instead analyzed the histological changes (at the same dose), reporting significant 76% reduction of gastric lesions area and increase of the repaired mucosa by 59% compared to the control group. Immunohistochemical analysis also revealed an increase in new blood vessels and increase of mucus in the mucosa glands [93]. Interestingly, in a more recent study, the same group highlighted the key role of β-myrcene, a minor component of *C. aurantium* EO, in granting protection from peptic ulcers [94]. Rats were induced with gastric ulcer by different methods including alcohol, indomethacin, cysteamine administration, stress, or by ischemia–reperfusion after two weeks of treatment with β-myrcene at a dose of 7.5 mg/kg. Results proved its anti-ulcer activity, with significantly decreased gastric and duodenal lesions as well as increased gastric mucus production. They also revealed significant modulation in activity of antioxidant enzymes through increase of glutathione reductase (GR) and peroxidase (GPx) and total GSH, accompanied by decreased activity of SOD and unaltered thioredoxin reductase (TrxR), demonstrating an increase in the levels of gastric mucosa defense factors by β-myrcene [94].

Myrtle (*Myrtus communis* L.) EO, containing myrtenyl acetate (30.6%), linalool (14.9%), α-pinene (11.10%), and 1,8-cineole (9.9%), microencapsulated with maltodextrin by emulsification and spray drying, was evaluated for the gastroprotective activity in ethanol/HCl-induced acute gastric ulcer in rats. Pretreatment with myrtle EO inhibited gastric lesions and acidity along with healing of lesions [95]. It expressed a potent anti-inflammatory effect on the gastric mucosa, counteracting lipoperoxidation and preventing the decline of the antioxidant enzyme activity (SOD, CAT, and GPx). Taken together, the gastroprotective action of the EO was attributed to its anti-inflammatory and antioxidant properties. The protection was dose-dependent, with a significant effect at 500 mg/kg b.w., while at 1000 mg/kg, the EO had similar efficacy as the drug famotidine (20 mg/Kg) [95].

Both sage (*Salvia officinalis* L.) and clary sage (*Salvia sclarea* L.) EOs have longstanding traditions in folk medicine for the treatment of gastrointestinal affections and ulcers. The antiulcerogenic activity of *S. officinalis* was confirmed in different studies and attributed to its antioxidant and anti-inflammatory properties [96]. The ethanol extract of *S. officinalis* leaves at doses of 100 and 150 μg/kg in rats, one hour prior to gastric ulcer induction, showed significant gastroprotection by reducing the total lesion area [97]. In a recent study, *S. officinalis* EO was shown to protect from indometacin-induced gastric ulcer in rats at a dose of 0.1 mg/kg b.w. [98].

*Achillea millefolium* L. belongs to the *Asteraceae* family and is commonly known as yarrow. It has traditional use in folk medicine in the form of tinctures and infusions as wound healing, as anti-inflammatory, to treat abdominal pain (e.g., menstrual contractions) and gastrointestinal symptoms, and to stimulate digestion [99,100,101]. A recent study demonstrated the gastroprotective action of yarrow essential oil against gastric ulcers induced in rats by extreme ethanol ingestion. Yarrow EO-pre-treated animals (100 or 200 mg/Kg b.w.) had fewer lesions, reduced ulcer surface, and reduced pro-inflammatory cytokines, apoptotic markers, and oxidative damage (MDA), while they had increased pH and pepsin and restored levels of gastroprotective prostaglandin PGE2, of GSH, and of antioxidant enzymes’ activities (SOD, CAT). The authors concluded that the gastroprotective effect is related to the anti-inflammatory action through the triggering of the Nrf2/HO-1 antioxidant pathway [85].

## 8. Essential Oils Useful in the Management of Metabolic Disorders

The combination of unbalanced Western diet (high-calorie and low-fiber food), chronic stress, and sedentary lifestyle favors the development of metabolic disorders such as metabolic syndrome, diabetes, obesity, and hyperlipidemia, which have grown enormously in the world population during last decades, particularly in high-density urban areas, representing a major health hazard in the modern world upon exclusion of communicable diseases. According to the International Diabetes Federation, diabetes prevalence was 8.8% of the world population in 2015 and is predicted to grow to 10.4% by 2040, while a global survey on obesity in 195 countries conducted in 2015 estimated over 600 million adults and over 100 million children [102]. Less-certain data are available on metabolic syndrome, whose prevalence is, however, estimated in about one-quarter of the global population [102]. Often, metabolic disorders are associated, and they build on each other, complicating the pharmacological treatment. Clearly, a dietary intervention is key to treat such kind of conditions, and not surprisingly, the search for health-oriented foods and nutraceuticals has received major impetus [103]. Many essential oils have shown beneficial properties, and some of them are also receiving clinical validation [104,105,106,107,108]. Those receiving the highest attention in recent literature are discussed in the following sections.

### 8.1. Essential Oils in Diabetes and Hyperglycemia

Inhibition of carbohydrate hydrolyzing enzymes α-amilase and α-glucosidase is a key target for oral hypoglycemic treatments for type 2 diabetes mellitus (T2DM). While pancreatic α-amylase hydrolyzes starch into oligosaccharides, these are further hydrolyzed by intestinal α-glucosidase into glucose, which is finally absorbed into systemic circulation, raising the plasma glucose level. In T2DM subjects with reduced glucose tolerance, postprandial plasma glucose rise can be smoothed by enzyme inhibitors, which slow down carbohydrate digestion, ameliorating their condition. In a comparative study on several terpenoid EO components, citral was the only one that showed mild inhibition of α-amilase compared to the reference drug acarbose. Instead, several monoterpenes (10 mM) showed inhibition of α-glucosidase with the following order of potency: (R)-(+)-limonene = (S)-(−)-perillyl alcohol > α-terpineol, while (R)-(+)-β-citronellol, terpinolene, citral, (R)-(−)-linalool, nerol, geraniol, and (S)-(−)-β-citronellol exerted weaker inhibition, and (L)-menthol and γ-terpinene did not show any significant α-glucosidase-inhibitory activity [107]. The same terpenes were tested for stimulation of glucose uptake in 3T3-L1 adipocytes, which represents another strategy to reduce glucose plasma levels. Geraniol, citral, limonene, and (R)-(+)-β-citronellol (1 μM) had the highest activity, while nerol, (S)-(−)-perillyl alcohol, γ-terpinene, and α-terpineol were weaker stimulants; and (S)-(−)-b-citronellol, terpinolene, and linalool did not affect the glucose uptake [86]. Thus, to a different degree, several terpenes of widespread presence in EOs, such as limonene, citral, and citronellol, have hypoglycemic activity, which helps rationalize the efficacy recorded for the whole oils. 

Many studies documented the potential of herbs and spices in ameliorating glycemic control in diabetes [105]. For several such herbs, studies are also available using the essential oils in translational animal models (drug-induced diabetic rats), and they have recently been reviewed [104]. The essential oil of *Citrus aurantifolia* (Christm.) Swingle (lime), containing (R)-(+)-limonene (*d*-limonene, 57.84%), neral (7.81%), and linalool (4.75%), among others, was administered at 100 mg/Kg b.w. to hyperglycemic rats for 14 days. It caused significant reduction in fasting blood and hepatic glucose, whereas hepatic concentration of glycogen was significantly increased [87]. Moreover, improvement in dyslipidemia was observed: serum concentrations of total cholesterol, triacylglycerol, and low-density lipoprotein-cholesterol LDL-C were significantly reduced, and high-density lipoprotein-cholesterol HDL-C was increased [109]. Fennel EO (*Foeniculum vulgaris* L.) effectively improved hyperglycemia by reducing fasting blood glucose (FBG) in diabetic rats when dosed at 30 mg/Kg b.w [104]. At the same dose, treatment of alloxan-induced diabetic rats with rosemary EO (*R. officinalis* L.) for 15 days afforded complete correction of all glycemic alterations and protected the liver and kidney from oxidative stress induced by the drug, fully restoring the depleted levels of reduced GSH, catalase (CAT), and superoxide dismutase (SOD) [110]. Similarly, lavender EO (*Lavandula stoechas* L.) at the dose of 50 mg/Kg b.w. showed reduction of blood glucose (FBS) and of lipoperoxidation owing to its antioxidant activity [104]. Myrtle EO (*Myrtus communis* L.) administered to alloxan-induced diabetic rats at the doses of 50 mg and 100 mg/Kg b.w. showed reduction of blood glucose (FBG) and of serum triglycerides and increase in glucokinase and liver glycogen; it also afforded the increase of antioxidant enzymes and decrease of the lipoperoxidation marker malondialdehyde MDA (TBARS, assay) [111]. Similarly, *Thymus vulgaris* EO in the same model reduced blood glucose while improving lipid profile at 40 mg/kg b.w. [104].

*Salvia spp.* EO certainly has interesting potential in the control of hyperglycemia. In the animal model, *Salvia sclarea* EO (clary sage) reduced blood glucose in the dose range 50–200 mg/Kg b.w. [104]. More interestingly, *Salvia officinalis* was studied in a double-blind, placebo-controlled clinical trial of type 2 diabetes mellitus (T2DM) patients who received three capsules of 150 mg/day for 3 months (Table 1) [112]. The study demonstrated a significant reduction of 2-h postprandial levels of hematic glucose (2hpp), while the reduction in fasting blood sugar (FBG) and glycosylated hemoglobin (HbA1c) did not reach significance, possibly requiring higher doses. The study also showed significant reduction of total plasma cholesterol (TC) and no sign of toxicity, indicating the beneficial and safe role of *Salvia officinalis* in diabetic patients [112].

Green cumin EO (*Cuminum cyminum* L.) certainly stands out for its efficacy in treating diabetes. The essential oil contains cuminaldehyde as the main component (42%) and *p*-cymene (16%), γ-terpinene (16%), and β-pinene (10%) as secondary components along with several (>20) other minor components [88]. Besides a number of studies on the animal model, a few very interesting clinical investigations are also available (Table 1) [113,114,115]. When administered to T2DM patients at 50 mg or 100 mg/day for 2 months, green cumin EO afforded large and significant amelioration of glycemic parameters [113]. Fasting blood glucose (FBG), glycosylated hemoglobin (HbA1c), and serum insulin were reduced, while insulin sensitivity (assessed by HOMA-IR index) was increased. During the treatment, the inflammatory factors high-sensitivity C-reactive protein (hsCRP), adiponectin, and TNF-α, which are normally related to insulin resistance, were all decreased. All charges were significant at both doses although the *p*-value was lower for patients who received the 100 mg supplement [113]. Another study (Table 2) at the lower dose of 25 mg/day vs. placebo and vitamin E supplementation (3 arms) reported similar findings, showing the superior efficacy of green cumin in the dietary intervention for diabetes compared to vitamin E, with improvement of both glycemic and lipidemic parameters, which are often dysregulated in T2DM patients, leading to increased cardiovascular risk [115]. In this regard, green cumin EO was also studied on prediabetic patients at the dose of 75 mg/day for 10 weeks, recording no significant alteration of FBG and HbA1c while showing significant increase in insulin sensitivity and important amelioration of lipidemic parameters [114]. LDL-C and leptin were reduced, while HDL-C was increased, with no significant variation of total cholesterol. Interestingly, body mass index (BMI) was reduced and total body weight (BW) and waist circumference (WC) were reduced more in female than in male patients, indicating green cumin EO as aid for weight control in women [114].

### 8.2. Essential Oils in Dyslipidemia, Metabolic Syndrome, and Obesity

High levels of circulating total cholesterol (TC), of low-density lipoprotein-cholesterol (LDL-C), and of plasma triglycerides along with low levels of high-density lipoprotein-cholesterol represent well-established risk factors for cardiovascular disease and might be associated with diabetes or dysregulated glycemic parameters in metabolic syndrome. Treating these unbalances with suitable dietary approaches is not less important than addressing hyperglycemia alone, and as seen in the previous section, some EOs, most notably green and black cumin, show parallel reduction of lipidemic factors along with glycemic (Table 1). Indeed, studies specifically addressing the amelioration of hyperlipidemia or the combined improvement of glycemic–lipidemic parameters in metabolic syndrome have become available in the literature, and representative clinical trials are summarized in Table 2.

A single-blind study on overweight and obese subjects receiving 3 g/day of green cumin (*Cuminum cyminum* L.) seeds powder for 3 months showed reduction in total serum cholesterol (TC) and triglycerides along with a decrease in HDL-C and increase in HDL-C, thereby improving all lipidemic parameters without any recorded side effect [120]. The result was confirmed in double-blind studies on T2DM patients receiving two different doses of green cumin essential oil: 30 mg/day or 100 mg/day for 2 months. With both supplement regimes, total serum cholesterol and LDL-C were significantly reduced; however, the higher dose also afforded significant increase in HDL-C [120]. Meta-analysis of those and other clinical trials concluded that the data demonstrate the beneficial effects of cumin supplementation on the control of serum/plasma lipid parameters; therefore, green cumin supplementation can be regarded as a safe therapeutic option besides statins to treat hyperlipidemia [120].

Black cumin (*Nigella sativa* L.) was also extensively investigated in this regard. A recent double-blind study involved obese and overweight healthy women who received cumin oil (2000 mg/day) or placebo for 8 weeks followed by a 4-week washout period. The supplement increased serum HDL-C and reduced LDL-C and the ratio TC/HDL-C (*p* < 0.001), which is considered the most important atherogenic risk factor. It also reduced serum glutamic-oxaloacetic transaminase (GOT, *p* < 0.05) and systolic blood pressure (*p* < 0.001), while there was no effect on diastolic blood pressure. Owing to overall improvements in cardiovascular risk factors, the study indicates beneficial effects of black cumin supplementation in adults with obesity [122].

Interestingly, another double-blind, cross-over clinical trial was conducted in patients with metabolic syndrome to assess the effect of consuming bread prepared with 3% black cumin seeds. The study lasted 3 months, during which patients received 100 g of bread/day (vs. control: regular handmade bread). No significant change in lipidemic or glycemic parameters was observed at the end [121], possibly because the active component of cumin EOs has been thermally degraded or lost by evaporation during baking. Indeed, the study reports no chemical analysis of the final product to verify this point.

Besides improving lipidemic parameters, it is interesting that several clinical trials investigated the role of *Nigella sativa* supplementation to reduce body weight in overweight subjects or in patients with metabolic syndrome, where weight control is important to improve the pathology. A recent meta-analysis evaluated 11 trials after filtering for quality parameters [123]. The meta-analysis concluded that *N. sativa* supplementation reduced body weight (−2.11 kg), body mass index (BMI) (−1.16 kg/m^2^), and waist circumference (WC) (−3.52 cm) significantly compared to placebo groups [123]. Therefore, supplementation with *N. sativa* can be regarded as a moderately effective and completely safe approach for weight control in overweight and metabolic syndrome subjects. No serious side effects were reported.

*Pistacia atlantica* L. subsp. *kurdica* (wild pistachio, *Anacardiaceae* family) was investigated for its activity against hyperlipidemia in T2DM patients in a triple-blind clinical trial in which randomized patients received a capsule of powdered dried fruits (500 mg/day) or of placebo for 2 months. After 1 or 2 months, postprandial glucose peak (2hpp), total cholesterol (TC), and LDL-C were reduced significantly (*p* < 0.05), but there was no statistically significant change in FBG, HbA1c, TG, HDL-C, alanine aminotransferase (ALT), aspartate aminotransferase (AST), and creatinine (Cr), which was attributed to the limited duration of the dietary intervention. Standardization of the plant materials by chemical analysis revealed 54 components of the essential oil fraction, which was considered the main bioactive fraction. The EO contained α-pinene (28.5%), bornyl acetate (8.7%), myrcene (5.6%), camphene (5.5%), and spatulenol (5.5%) as the main components [124]. 

*Melissa officinalis* L. (lemon balm) essential oil is rich in neral, nerol, geranial, citronellal, citronellol, geranyl acetate, and β-caryophyllene as the main (characteristic) components [128]. Some of such terpenes have been shown to express anti-lipidemic activity, e.g., in cultures of 3T3-L1 adipocytes [108]. It was recently reported that *M. officinalis* significantly improves lipidemic profile in T2DM patients who received 1.4 g/day of hydroalcoholic extract for 8 weeks, with increase of Apo A-I and decrease of TC/HDL-C ratio and of LDL-C/HDL-C ratio (Table 2) [125]. However, it is unclear how much of the effect is attributable to EO components, which were not analyzed in the extract.

Garlic (*Allium sativum* L.) is a widely consumed food with various medicinal properties, including a strong reputation as an anti-hypertensive, anti-hyperlipidemic food, which protects from cardiovascular diseases and metabolic syndrome. Studies on the animal model support these expectations, showing the protective properties of garlic EO and its major organosulfur component (diallyl disulfide), particularly dose-dependent anti-obesity and antihyperlipidemic effects by reducing drug-induced body weight gain, adipose tissue weight, and serum biochemical parameters [129]. In a recent clinical trial on T2DM patients with blood cholesterol > 200 mg/dL, oral intake of a soft capsule containing garlic EO 13.5 mg/day for 6 months resulted in significant reduction of fasting blood glucose, HbA1c, serum creatinine, and blood cholesterol (TC) at the third and sixth months. The study concluded that *Allium sativum* essential oil may prove helpful in treating hypercholesterolemia as an adjunctive therapy or for those intolerant to statin therapy [126].

Other essential oils have accumulated scientific evidence supporting their efficacy in ameliorating dyslipidemia through studies in the animal model or in vitro, supported by robust rationale. These include fennel (*Foeniculum vulgare* Mill.), sweet orange (*Citrus aurantium* L.), sage (*Salvia officinalis* L.), and particularly grapefruit (*Citrus grandis* Osbeck), which has been extensively investigated for its anti-obesity potential [107]; however, they have not yet received clinical validation.

Black cumin (*Nigella sativa* L.) is perhaps the aromatic plant that has received the highest attention in recent years, and many studies, including several clinical trials, have become available on its efficacy against hyperglycemia. A selection is summarized in Table 1. *Nigella sativa* seeds powder administered 2 g/day or 3 g/day (but not 1 g/day) for 3 months in T2DM patients showed significant improvement of all glycemic parameters. It caused significant reductions in FBG, 2hpp, and HbA1c without significant change in body weight. Insulin resistance calculated (by HOMA2) was reduced significantly (*p* < 0.01), while β-cell function was increased (*p* < 0.02). The use of *Nigella sativa* in a dose of 1 g/day also showed trends in improvement, but these were not statistically significant. However, no further increment in the beneficial response was observed with 3 g/day vs. 2 g/day dose. The doses of *Nigella sativa* used in the study did not show adverse effects [116]. 

Parallel results were obtained in another study on T2DM patients with 2 g/day cumin seed powder administered for 1 year, which showed no significant toxicity and highly improved glycemic parameters (Table 2). The study also demonstrated an increased plasma total antioxidant capacity (TAC) and increased antioxidant enzymes superoxide dismutase (SOD) and catalase (CAT), with raised levels of GSH and decreased markers of lipoperoxidation (TBARS) [117]. Two other clinical trials involved for 3 months T2DM patients who received *Nigella sativa* oil instead of the seed powder (Table 1). One administered 2.5 mL oil (2.3 g)/day and the other 2 g oil/day, selectively involving patients referred to a dialysis center [118,119]. Both obtained a consistent outcome that also confirmed that of the other studies: a significant reduction of FBG (and 2hpp [118]) and HbA1c. One study also observed reduction of body mass index [118], while the other recorded an increase in antioxidant enzymes and a reduction of lipoperoxidation [119]. Overall, the above studies and others fully demonstrated the potential and suitability of *Nigella sativa* in the treatment of T2DM pathology and the amelioration of dysregulated glycemic conditions, without any significant side effect even after one year of continuous intake. One point, however, needs to be addressed concerning the effective dose. Although the investigated doses were apparently high, ranging 2–3 g/day, it should be noted that all studies used the powdered seeds or the seed oil as the source of essential oil (the active part rich in timoquinone). Since the oil is mainly composed of triglycerides and contains only about 2% EO, while the seed contains about 20% oil [127], it can be estimated that 1 g of seeds powder corresponds to 4 mg EO, while 1 g of oil corresponds to 20 mg EO. Therefore, the posology used in the trials likely corresponds to 8–50 mg/day of EO.

## 9. Essential Oils to Protect Liver Function and Stimulate Digestion

In classical aromatherapy several essential oils are used to stimulate digestion, among them clary sage (*Salvia sclarea* L.) lemon (*Citrus limon* L.), coriander (*Coriandrum sativum* L.), ginger (*Zingiber officinalis* L.), and, most notably, rosemary (*Rosmarinus officinalis* L.). This has been attributed at least in part to interaction of EO components with receptors for specific types of “taste” in the gastrointestinal tract, such as bitter taste TAS2R receptors and pungent taste transient receptor potential vanilloid receptors TRPV1 or others, such as ankyrin subtype 1 TRPA1 or TRPM8 [130]. Such interaction would activate digestive functions such as bile secretion, gastric motility and secretions, secretion of gastric protective mucus, pancreatic digestive enzymes, etc., thereby promoting all digestive functions by activating the neuroendocrine machinery [130]. Several preclinical and clinical studies supporting these finding have already been reviewed [130]. Besides stimulating digestion, EOs have been recently fount to protect digestive functions and organs from inflammatory and oxidative-stress-related damage. In particular, the liver is subject to major metabolic attack by radicals produced as a consequence of phase-1 detoxifying enzymes activation by pollutants, toxins, and xenobiotics.

*Salvia officinalis* L. EO was recently demonstrated to exert hepato/renal protection in mice from damage induced by carbon tetrachloride administration: a well-established model for induced massive radical damage in liver [131]. Administration of sage EO at 0.1 to 0.4 mL/Kg b.w. showed reduction of liver enzymes (ALT, AST, ALP LDH, and G-GT) induced by CCl_4_; it lowered bilirubin, urea, and creatinine along with increasing total protein, albumin, globulin, and prothrombin. It also showed a decrease in lipoperoxidation and DNA breakage levels along with repairing histo-architectural distortions. The efficacy was attributed to activation of antioxidant defenses by the essential oil [131].

*Rosmarinus officinalis* L. was evaluated in a similar experimental model against CCl_4_-induced hepatotoxicity in rats. At the dose of 5 mg and 10 mg/Kg b.w., it afforded reduction of transaminases ALT and AST in serum up to 2-fold, it counteracted the increase in lipoperoxidation induced by CCl_4_, and it increased the activity of antioxidant enzymes catalase (CAT), superoxide dismutase (SOD), glutathione peroxidase (GTx), and glutathione reductase (GR), which was possibly the mechanism for the displayed hepatoprotection [132]. In a recent study, *R. officinalis* EO formulated in nanoemulsion afforded protection in rats against thioacetamide induced hepatic injury, significantly counteracting the rise in transaminase enzymes induced by the toxicant [133].

*Carum carvi* L. (caraway), in thioacetamide-induced hepatic injury in rats, exhibited significant restoration of levels of SGOT, SGPT, ALP, bilirubin, triglycerides, lipid peroxidation, GSH, and albumin [134].

## 10. Essential Oils for Diuresis and Protection of the Urinary Tract

Caraway (*Carum carvi* L.) is one of the oldest spices traditionally cultivated in Europe and then diffused in India and in North and Central America. The EO typically contains cuminaldehyde (17%), γ-terpinene (31%), limonene (16%), α-pinene (5%), β-pinene (4%), and *p*-cymene (7%) as the main terpenoid components. It has both acute and chronic diuretic activity that are well-documented in animal studies. In rats, acute administration of one dose 5 mL/Kg b.w. per os showed significant increase in diuresis and of electrolyte secretion (Na^+^, K^+^) without variation of creatinine levels. Under chronic regime, 10 mg/Kg b.w. for 8 days resulted in significantly increased diuresis without variation of electrolytes secretion or other parameters [134]. Caraway EO also has nephroprotective activity as demonstrated in experimental nephropathy in rats. Diabetic nephropathy was induced by the subcutaneous injection of STZ (60 mg/Kg). *Carum carvi* EO (10 mg/Kg) administered orally for 21 days significantly counteracted the increased levels of blood glucose and increased the level of GSH. It also counteracted kidney glomerular and tubular degeneration and hemorrhage in interstitial tissue [134].

Fennel (*Foeniculum vulgare* Mill.) has a longstanding tradition as a diuretic, anti-inflammatory, and antimicrobial aid to protect the urinary tract. The diuretic effect of the EO and particularly the anti-inflammatory effect has been documented in animal studies, while several microbiological studies highlighted its activity against *S. aureus* and *C. albicans* [135]. The combination of these bioactivities makes it particularly suited for the health of the urinary tract.

Juniper (*Juniperus spp*.) is often used as adjuvant in the protection of urinary tract because of its anti-inflammatory activity associated with selective antimicrobial activity on the pathogen strains that colonize the tract. The recent investigation of the EOs from three *Juniperus* species (*J. communis*, *J. horizontalis*, and *J. chinensis*) indicated that *J. horizontalis* oil has the highest activity against *E. coli*, while *J. communis* has the highest activity against *S. aureus*. The three oils were found to significantly decrease the production of the pro-inflammatory cytokines tumor necrosis factor (TNF)-α, interleukin (IL)-1β, and gamma interferon (INF-γ) in lipopolysaccharide-activated white blood cells. *J. chinensis* oil possessed the highest potency against IL-1β. Analysis afforded the identification of 45 components, and it was assessed that that 1-terpineol, 4-terpineol, bornyl acetate, limonene, and α-pinene are the positive contributors to both bioactivities (antimicrobial and anti-inflammatory), while β-thujone, 3-carene, and γ-muurolene contributed to IL-1β inhibitory activity [136]. Juniper EO was also shown to have selective activity against mycotic dermatophyte, *Aspergillus*, and *Candida* strains that often colonize the urinary tract. A study evaluated *J. communis* ssp. *alpina*, *J. oxycedrus* ssp. *oxycedrus* and *J. turbinate* [137]. Antifungal activity was evaluated on clinical and type strains of *Candida*, *Aspergillus*, and dermatophytes. The oil from leaves of *J. oxycedrus*, which was particularly rich in α-pinene (65.5%) and δ-3-carene (5.7%), was the most active, with MIC and MBC values ranging from 0.08–0.16 μL/mL to 0.08–0.32 μL/mL, respectively [137]. 

## 11. Essential Oils to Reduce Inflammation and Pain

Inflammation is part of our immune response, and it is actually necessary to trigger it. It is a complex regulated process, and in principle, it should terminate when the cause, e.g., the invasion by a pathogen, has been eliminated. However, loss of regulation occurs not infrequently, leading perhaps to more danger than that which triggered it. It can lead to the development of chronic disease such as asthma, atherosclerosis, inflammatory bowel disease, cardiovascular diseases, neurological disorders, and cancer [138,139]. Inflammation is usually classified into two categories according to its time course and intensity: acute inflammation and chronic inflammation. The two types are characterized by different involvement of immune cells: Acute inflammation of a tissue is often associated with infiltration by innate lymphoid cells such as macrophages and neutrophils, while chronic inflammation is instead often associated with tissue infiltration by more specialized T cells and plasma cells [139]. Although both steroidal drugs (e.g., cortisol and mimics) and non-steroidal anti-inflammatory drugs (NSAID) are abundantly available, they are not free from side effects, and their long-term administration, e.g., in chronic inflammation, may not be the best solution. Many plant essential oils have shown excellent efficacy in this regard, with a generally safer profile, which makes them ideal candidates for alternative therapeutic approaches such as dietary interventions enriched by EO-based nutraceuticals [139]. Several reviews have analyzed the literature concerning anti-inflammatory essential oils [139] and their monoterpene components [55,140], sesquiterpene components [141,142], and phenylpropanoid components [143]; therefore, we summarize here only some notable examples, and we refer to such works for deeper discussion. EO components such as thymol, thymoquinone, 1,8-cineol, fenchone, α-pinene, β-pinene, citronellol, linalool, myrcene, carvacrol, limonene, menthol, *p*-cymene, α-phellandrene, α-terpineol, terpinen-4-ol, caryophyllene, bisabolol, chamazulene, δ-3-carene, cinnamaldehyde, eugenol, methyleugenol, and anethole were shown effective in reducing paw edema in rats or were active in other in vivo inflammation models at doses equal to or lower than 25 mg/kg b.w. Particularly noteworthy are 1,8-cineol, fenchone, β-pinene, and thymoquinone, which were active at doses equal or lower than 1 mg/Kg b.w., i.e., showing efficacy of the same magnitude as NSAID drugs such as diclofenac [140,141,144]. The mechanism of anti-inflammatory action is often related to a decrease of the level of pro-inflammatory cytokines such as TNF-α, IL-1β, IL-4, IL-5, IL-6, and IL-8; to the decreased expression of lipoxygenase (LOX) and cyclooxygenase (COX); or to inhibition of their activity, with reduction of pro-inflammatory leukotrienes and prostaglandins formed by these two enzyme systems from arachidonic acid to inhibition of myeloperoxidase (MPO) and with reduction of oxidative stress to the induction of antioxidant enzymes (e.g., CAT, SOD, GPx, GR) that counteract inflammation-related oxidative stress and tissue damage, thereby blocking the vicious circle of radical production–tissue damage–inflammation [139,140,141,142,144]. Additionally, modulation of nitric oxide synthase NOS with regulation of the blood vessels’ lumen is sometimes registered. (*E*)-1-(3,4-dimethoxyphenyl) butadiene (DMPBD) found in *Zingiber cassumunar* Roxb. EO was extensively tested in vitro and in vivo in various models of experimental inflammation. DMPBD dose-dependently inhibited the rat ear edema induced by ethyl phenylpropiolate (EPP), arachidonic acid (ArA), and 12-O-tetradecanoylphorbol 13-acetate (TPA), and it was more potent than any tested standard drugs such as diclofenac, oxyphenbutazone, and phenidone [145]. It had equal activity to diclofenac in inhibiting the carrageenan-induced rat paw edema. The potent activity of DMPBD occurs through inhibition of both cyclooxygenase (COX) and lipoxygenase (LOX) pathways [146]. The above evidence justifies the potent anti-inflammatory activity of EOs rich in such components as eucalypt, niaouli, black cumin, thyme, oregano, savory, rosemary, blue (*Matricaria*) chamomile, lavender, peppermint, anise, fennel, clove, cinnamon, lemongrass, lemon balm, Cassumunar ginger, *Citrus* spp., *Pinus* spp., *Abies* spp., and *Juniperus* spp. [139,140,141,142,144,146].

These biological activities make EOs useful in treating chronic inflammatory pathologies such as arthritis and different forms of pain, explaining their use in traditional Chinese medicine [139] and in aromatherapy [143]. For example, clinical trials proved the efficacy of lavender EO, peppermint EO, *Matricaria chamomilla* EO, anise EO, basil EO, rose EO, and garlic EO in treating headache and migraine [143].

Clary sage EO [145] and particularly lavender EO [147] have proven effective in treating primary dysmenorrhea in controlled clinical trials, which is attributed to the muscle-relaxing as well as anti-inflammatory activities. Moreover, *Thymus vulgaris* and *Zataria multiflora* Boiss., a plant native of southwest Asia belonging to the *Laminaceae* family, whose EO contains thymol and carvacrol as main and second main components, were shown effective in reducing pain associated to dysmenorrhea in clinical trials [143].

## 12. Essential Oils with Immunomodulatory and Anti-Influenza Activity

Anti-inflammatory drugs such as NSAIDs are often used to alleviate the symptoms associated to cold-season illness and influenza. However, they do not counteract or prevent the viral infections causing them. Essential oils can instead be a particularly useful alternative or addition, as they are associated with potent anti-inflammatory activity (see previous section), immune-stimulating activity, and, in some cases, also with antiviral activity.

A recent literature survey concerning the immunostimulant activity of EOs pointed out some differences among studies depending on their type, distinguishing in vitro, pre-clinical, clinical, or those based on diet supplementation of animals. It suggested that further studies are needed to clarity the matter although a few EOs were found to stand out in all families of studies: eucalypt and ginger [148]. However, other EOs have accumulated evidence for their efficacy in many studies, including human; these are thyme, lavender, clove, tea tree, and citruses (lemon, orange, and bergamot) [138]. The mechanism of immune stimulation is often complex to rationalize, as the same EO typically also expresses anti-inflammatory activity through the down-regulation of pro-inflammatory cytokines, and these, in principle, should stimulate immune response. Although the modulation varies with the actual EO and, even within the same botanical species, with the actual composition of the EO used in the study (i.e., with the chemotype), often, immune stimulation is linked to boosting innate immune response, and it is associated with interfering with the NFkB, p38, or ERK/MAPK signaling pathways [138]. Eucalyptus EO, with prevalent 1,8-cyneole content, was found to increase phagocytosis by inducing monocyte-derived macrophages with increased phagocytic activity. The activity of tea tree oil rich in tepinen-4-ol was attributed to activation of NF-kB factor, which increases phagocytic activity. Both tea tree oil and terpinen-4-ol induce the differentiation of immature myelocytes into active phagocytizing monocytes and increase the expression of CD11b, a receptor that is partially responsible for the phagocytosis of opsonized bacteria and fungi by leukocytes [138].

Selected EOs demonstrated antiviral activity against influenza viruses in vitro; for instance, *Citrus reshni* (limonene 82% and linalool 7%) had EC_50_ = 2.5 μg/mL against influenza virus A (H5N1), *Cinnamomum verum* (main component cinnamaldehyde) inhibited influenza A/PR/8 virus in vitro (at 200 μM) and increased survival of inoculated mice in vivo, and *Curcuma aeruginosa* (rich in germacrone) had EC_50_ = 6 μM against influenza A/H1N1/H3N2 virus strains and was active against the influenza B virus in dose-dependent manner [149]. *Melaleuca alternifolia* (tea tree) oil (terpinen-4-ol 37%, γ-terpinene 22%, and α-terpinene 10%) had antiviral activity (ID_50_ = 0.0006% *v*/*v*) against the influenza A⁄PR⁄8 virus subtype H1N1, with activity attributed to terpinen-4-ol [149]. *Pogostemon cablin* Benth. (patchouli) oil had in vitro activity (IC_50_ of 4.0 μM) against influenza virus A (H2N2) and afforded significant increase in the survival of infected mice. *Salvia sclarea* (linalyl acetate 61%, linalool 22%) had inhibitory activity (>52%, IC_50_ < 100 μg/mL) against influenza A/WS/33 virus-infected MDCK cells, increasing cell survival [149]. Coriander and ginger were found effective in treating different respiratory tract infections by bacteria and viruses. Among the latter, coriander showed inhibition against viruses such as dengue and Middle East respiratory syndrome (MERS) coronavirus [150]. Efficacy was explained by the binding of aldehyde components of the oil (such as 2-(*E*)-dodecenal) to non-structural proteins of the virus [150]. Ginger has instead been reported to inhibit the growth of SARS-CoV-2 coronavirus by blocking the receptor of SARS-CoV-2/TMPRSS2 due to interaction with gingerol [150]. Interestingly, one randomized clinical trial found that *Thymus vulgaris* EO as 5 mL dispersed in a sweet syrup and administered 3 times a day (dose = 14 g/day) in COVID-19 patients for one week afforded significantly reduced fever (*p* < 0.027), dizziness (*p* < 0.003), cough, dyspnea, muscular pain, headache, anorexia, weakness and lethargy, fatigue, and chest wall pain (*p* < 0.001). In addition, blood urea nitrogen (*p* < 0.004), neutrophil count (*p* < 0.001), and calcium were (*p* < 0.034) decreased, but lymphocyte count was increased significantly (*p* < 0.001) [151].

## 13. Essential Oils for Neuroprotection and Modulation of Mood and Cognitive Function

With the global increase of life expectancy, the prevalence of age-related diseases is also increasing, particularly including neurodegenerative conditions such as Alzheimer’s disease (AD). Neurodegenerative diseases and other chronic inflammatory conditions characterized by cognitive impairment are related to lifestyle and to the diet; hence, a dietary intervention can help their course and patients’ quality of life. In AD, the two main microscopic hallmarks of disease, the abnormal accumulation of extracellular protein material in the amyloid–beta plaques and the formation of intracellular neurofibrillary tangles, are associated with abnormal expression of acetylcholinesterase (AChE), which leads to decreased levels of neurotransmitter acetylcholine (ACh), synaptic alteration, and impaired memory and learning [152]. Increased AChE expression appears to be related both to the formation of plaques and to the impaired cognitive function; hence, inhibiting AChE is currently the main pharmacological strategy to treat AD [152,153]. On the other hand, research efforts are clarifying the association between AD, inflammation, and oxidative stress, outlining that antioxidants are key to effective AD treatment strategies [153,154,155]. One additional point is the association between AD and impaired glycemic control, with increased incidence of T2DM in patients with dementia [152]. The above constitutes a solid rational basis to understand the potential of dietary EO supplementation in protecting from neurodegenerative conditions such as AD because (1) many EOs have excellent anti-inflammatory activity with low incidence of side effects; (2) many EOs have good antioxidant activity; (3) many EOs are effective in improving glycemic control and T2DM parameters; and (4) EOs are small, lipophilic molecules with high diffusivity, and hence, they have excellent ability to cross the blood–brain barrier and reach the central nervous system. A fifth reason is that some EOs [156,157] and EO components [156,157,158] have been demonstrated to possess good inhibition activity toward AChE, thereby representing a promising aid to tread AD and other cognitive-impairment conditions [158]. Therefore, several essential oils have the potential to ameliorate the cognitive function and protect from AD; these include thyme, sage, eucalypt, lemon balm, peppermint, oregano, rosemary, lavender, basil, and citrus EOs (rich in limonene and citral) owing to the combination of glucose-lowering, anti-inflammatory, antioxidant, and anti-AChE activities [152,156,157,158].

*Origanum majorana* (L.) EO has recently been demonstrated to improve the cognitive function in Alzheimer’s amyloid beta1–42 rat model owing to the combined antioxidant activity and enhanced brain-derived neurotrophic factor (BDNF) expression, which enhances the cognitive function [159]. *Salvia officinalis* (L.) EO is regarded as one of the most promising natural remedies for AD on the basis of numerous animal studies, in which it showed to enhance memory and cognitive function in rats also by expressing anti AChE activity, to have neuroprotective activity due to boosting the antioxidant defense machinery in vivo, and to have anti-seizure effects [160]. This was confirmed in clinical investigation [160]. A study addressed the efficacy of the essential oils of the *Salvia* species to affect cognition and mood in 135 healthy adults. It was demonstrated that both *Salvia officinalis* and *Salvia lavandulaefolia* EOs are capable of modulating cognition and mood. Cognitive performance assessed via the Cognitive Drug Research (CDR) System indicated that the *S. officinalis* group performed significantly better than the control group, while the alert mood measure displayed significant differences between both EOs and the control [161]. In a 4-month, parallel group, placebo-controlled clinical trial undertaken in three clinical centers, patients with mild to moderate AD aged between 65–80 years and treated with an alcoholic extract of *S. officinalis* leaves showed significant improvement in the cognitive subscale of Alzheimer’s Disease Assessment Scale (ADAS-cog) and in the clinical dementia rating (CDR), demonstrating the efficacy of *S. officinalis* extract in the management of mild-to-moderate Alzheimer’s disease [162].

Bergamot (*Citrus x bergamia,* Risso & Poit.) is perhaps the essential oil that is accredited the highest potential for complementary AD treatment in most recent research [163]. This EO offers extensive preclinical evidence of analgesic and anxiolytic properties needed to counteract the behavioral and psychological symptoms of dementia (BPSD). Additionally, such symptoms are found to involve glutamatergic neuronal transmission, and bergamot EO is found to increase synaptic glutamate [163]. A clinical trial on 134 patients with severe dementia is planned [164]. Other promising EOs in this regard are lavender and lemon balm (Table 3).

Modern aromatherapy is using essential oils to treat anxiety, stress, panic and phobia, and other forms of emotional discomfort. Building on evidence in animal studies, a significant body of research is now being dedicated to unambiguously prove with controlled, randomized clinical trials the efficacy of EOs to treat such mood disorders [183]. Several clinical trials have already provided evidence of the efficacy of some EOs for this purpose, and a representative selection of such studies is summarized in Table 3.

For instance, *Melissa officinalis* L. (lemon balm) EO was tested in acute coronary syndrome (ACS) patients, resulting in significantly reduced scores of stress and anxiety, with reduced heart rate (*p* < 0.001) along with remarkable decrease in the mean arterial pressure in the Melissa group compared to the placebo group (*p* < 0.001) [176]. *Lavandula angustifolia* L. (lavender) EO was tested for 4 weeks on patients with insomnia, showing significantly improved sleep quality and duration, improved quality of life, and improved overall mood [178], while the combination of lavender with chamomile (*Matricaria recutita*) showed statistically significant improvement in depression, anxiety, and stress levels immediately and one month after the intervention in the lavender and chamomile group compared to the control group (*p* < 0.01) [171]. Similarly, lavender combined with Roman chamomile (*Anthemis nobilis*) and neroli (*Citrus aurantium* flowers EO) showed reduced anxiety and improved sleep quality [181]. 

Of interest, certain essential oils were found effective in alleviating fatigue and giving tone and could be useful to counteract mental fatigue for students or physical fatigue for recovery after sport and exercise or during convalescence from illness. 

For instance, either *Mentha piperita* (peppermint) or *Lavandula angustifolia* (lavender) dispersed in water or placebo (just water) were administered by inhalation (3 drops/night) for 7 nights to 105 hospitalized cardiac patients, recording the fatigue severity scale (FSS) before and after the intervention. Both the lavender and peppermint groups recorded significantly decreased average fatigue that was statistically different from control (*p* < 0.001), but there was no significant difference between the two EO groups [174]. 

Another study on healthy volunteers (physical exercise students) showed that inhalation of either *Citrus sinensis* L. (sweet orange) EO or *Mentha spicata* L. (spearmint) EO before a distance run resulted in significantly decreased run times in both EO groups. Moreover, a lung function tests showed an improvement in the lung status for the students after inhaling the oils, with a significant increase in forced expiratory volume in the first second and forced vital capacity after inhalation for the both oils [175]. Similar results were obtained in a similar study using 0.05 mL peppermint oil (*M. piperita*) dispersed in mineral water [184]. These studies reinforce pre-clinical evidence of markedly decreased fatigue in exhaustive exercise by administration of lemon peel oil (*Citrus limon* L.) [185].

It should be noted that in most trials shown in Table 3, EOs were administered to patients or volunteers by inhalation, as this is regarded as the preferred way in modern aromatherapy. However, it is useful to recall that classical aromatherapy instead used oral intake as the preferred form, and conceivably, most if not all the outcomes resulting from studies based on EO inhalation can be translated into oral intake, which is compatible with nutraceuticals and diet supplements upon identifying the equivalent dose.

## 14. Conclusive Remarks and Perspective

Analysis of the literature might raise some puzzling questions about the fact that the same essential oil can have different, sometimes even apparently opposite bioactivities. This is partly explained by the fact that these actions are underpinned by the modulation of common biochemical processes, such as the modulation of inflammatory cytokines or the induction of antioxidant enzymes, as we have tried to outline in the previous sections. However, another critical aspect needs to be considered: the chemotype of the tested EO.

As anticipated (Section 2), EOs from the same botanical species can have very different composition; hence, they are only nominally “the same essential oil”. For instance, 20 chemotypes of *Thymus vulgaris* EO have been described [186], and the difference among them can be as large as the difference from the EO obtained from other species (e.g., *Origanum vulgare* [20]). Large diversity is known for basil EO (*Ocimum* spp. count > 130 chemotypes with 76 belonging to *O. basilicum*) [187], chamomile [188], sage [189], rosemary, and for other EOs, with clearly different biological activity [186,189]. Therefore, it would be important to identify the chemotype of the tested EO when studying its biological activity, a feature often overlooked in the biomedical literature and that we warmly encourage. Alternatively, the actual composition of the EO specimen must be considered.

To express their healthy functional role in food products, molecules should have sufficient oral bioavailability. Essential oils are composed of small lipophilic molecules with high diffusivity; therefore, their absorption in the GI tract following oral intake is normally assumed as nearly quantitative or regarded as a non-critical issue [190]. Limited data are available on the pharmacokinetics in humans; however, studies in rats on radio-labelled ^14^C-citral and ^14^C-*trans*-anethole, a terpenoid and a phenylpropanoid, respectively, indicate a rate of GI absorption of 91–95%, and similarly high bioavailability was reported in human studies with unlabeled terpenoids [190]. Owing to their physical-chemical properties, EOs and their components can easily be dissolved in the lipid matrix of food products or incorporated in the lipid phase of emulsified products, while a surfactant with high hydrophilic/lipophilic balance (HLB > 15) would allow their micellar dispersion into aqueous products. In the manufacturing of diet supplements, they can easily be dissolved in a vegetable oil and incorporated in soft-gel capsules or be adsorbed in “inert” minerals such as CaCO_3_ or silica or in “inert” organic powders such as maltodextrins, and the compost can be incapsulated in hard-gel capsules [10]. However, two critical points need to be considered in formulation: their volatility (and limited stability), which requires the avoidance of heating processes, and their flavor/aroma. This last is paradoxically the main criticism associated with the use of EOs as functional ingredients in food products, and it intersects with another critical point: the effective dose. Indeed, the dose level requested for functional bioactivity can be quite high, ranging from tens of mg/day to over one g/day, as can be inferred from previous sections, which might result as excessive considering the intensity of their flavor, particularly if it is not “in taste” with the local dietary habits. These issues can sometimes be handled by an appropriate selection of the EO; however, encapsulation of the EOs can offer advantages in this regard, as it could smooth the perception of their flavor, particularly when they are dosed in gastro-resistant soft/hard-gel capsules or formulated in enteric micro- or nano-capsules [191]. Incorporation of EOs in nanoemulsions or in micro-nano-capsules might also increase their stability and bioavailability [191]. Since absorption occurs mainly in the small intestine [190], one additional advantage of gastro-resistant formulations is that they can deliver the EO where most of the absorption occurs, maximizing its efficacy and allowing a reduction of the effective dose. For instance, gastro-resistant forms of peppermint EO afforded clinical efficacy at a lower dose compared to conventional formulations in the treatment of IBS [37] (see Section 4).

Of interest, clinical studies highlighted that, at the tested doses, all the tested EOs had negligible incidence of side effects and were well-tolerated by volunteers.

In conclusion, plant essential oils are easily accessible, highly bioavailable, well-perceived natural sources of functional molecules for health-oriented functional food and nutraceuticals, whose distinctive advantages fully deserve the interest they are receiving.

**Figure 3:**
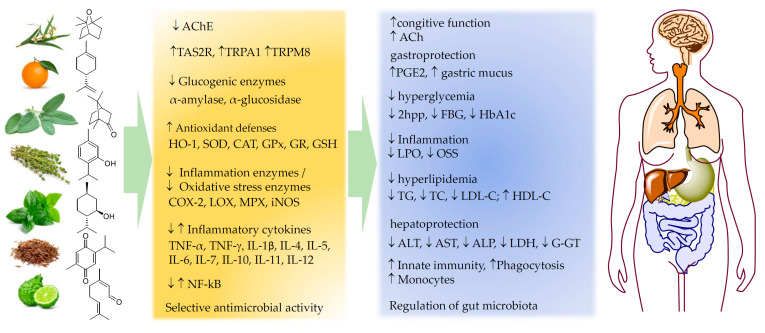
Summary of the biochemical interactions of EO components inducing physiological responses and ameliorating pathological conditions (↑ or ↓, increase or decrease of initial condition).

A summary of the actions of EOs on human physiology is provided in Figure 3.

## Figures and Tables

**Table 1:** Representative clinical trials of essential oils in the treatment of hyperglycemia.

Plant	Treatment	Study model	Effects	Ref.
Sage *(Salvia officinalis)*	(150 mg × 3)/day; 3 months	T2DM patients, DB	↓↓2hpp; (NS)FBS; (NS)HbA1c; ↓↓TC	[112]
Green cumin (*Cuminum cyminum*)	50 mg or 100 mg/day; 2 months	T2DM patients, DB	↓↓FBG; ↓↓HbA1c; ↓↓insulin; ↓TNF-α; ↓hsCRP; ↑↑adiponectin; ↓HOMA-IR	[113]
Green cumin (*Cuminum cyminum*)	75 mg/day; 10 weeks	Prediabetic patients, DB	↓↓HOMA-IR; (NS)FBS; (NS)HbA1c; ↓LDL; ↑HDL; ↓↓leptin ↓WC; ↓BMI; ↓BW	[114]
Green cumin (*Cuminum cyminum*)	25 mg/day; 3 months	T2DM patients, DB vs. Vit. E	↓↓FBG; ↓HbA1c; ↓oxLDL; ↓ApoA1; ↑↑paraoxonase 1; ↓↓leptin; ↓↓TG	[115]
Black cumin (*Nigella sativa*)	2 g seed powder/day; 3 months	T2DM patients, DB	↓2hpp; ↓↓FBG; ↓HbA1c; ↓↓HOMA2-IR; ↑↑β-cell	[116]
Black cumin (*Nigella sativa*)	2 g deed powder/day; 1 year	T2DM patients, DB	↓2hpp; ↓FBG; ↓HbA1c; ↓HOMA2-IR; ↑↑β-cell; ↑↑CAT; ↑SOD; ↑↑GSH; ↓↓TBARS	[117]
Black cumin (*Nigella sativa*)	2.5 mL oil/day; 3 months	T2DM patients, DB	↓2hpp; ↓FBG; ↓HbA1c; ↓BMI;	[118]
Black cumin (*Nigella sativa*)	2 g oil/day; 12 weeks	T2DM dialysis patients, DB	↓FBG; ↓HbA1c; ↑↑insulin; ↓hsCRP; ↑SOD; ↑TAC; ↓TBARS	[119]

2hpp, 2-h postprandial blood glucose; HbA1c, glycosylated hemoglobin; FBG, fasting blood glucose; HOMA2-IR, insulin resistance index; hsCRP, high-sensitivity C-reactive protein; BMI, body mass index; WC, waist circumference; BW, body weight; TC, total plasma cholesterol; TAC, total antioxidant capacity; NS, not statistically significant; DB, double-blind study; T2DM, type 2 diabetes mellitus; ↑ or ↓, variation within +/– 20% the initial value; ↑↑ or ↑↑, variation > 20%; LDL, low density lipoproteins; oxLDL, oxidized LDL; ApoA1, apolipoprotin A1, TG, plasma tryglycerides; SOD, superoxide dismutase; GSH, glutathione; TBARS, thiobarbituric acid reactive species (MDA).

**Table 2:** Representative clinical trials of essential oils in the treatment of dyslipidemia.

Plant	Treatment	Study Model	Effects	Ref.
Green cumin (*Cuminum cyminum*)	3 g seeds powder/day; 3 months	Overweight and obese, SB	↓TC; ↓↓TG; ↓LDL-C; ↑HDL-C	[120]
Green cumin (*Cuminum cyminum*)	100 mg EO/day; 2 months	T2DM patients, DB	↓TC; ↓LDL-C; ↑HDL-C	[120]
Green cumin (*Cuminum cyminum*)	50 mg EO/day; 2 months	T2DM patients, DB	↓TC; ↓LDL-C	[120]
Black cumin (*Nigella sativa*)	100 g bread with seeds/day; 2 months	Metabolic synd. patients, DB	No significant change in lipid profile	[121]
Black cumin (*Nigella sativa*)	2 g oil/day; 2 months	Overweight and obese women, DB	↓TC/HDL-C; ↓TG; ↓LDL-C; ↑HDL-C; ↓GOT; ↓SBP; (NS)DBP	[122]
Black cumin (*Nigella sativa*)	100 mg extract to 3g oil/day	Meta-analysis of 11 clinical trials	↓BMI; ↓BW; ↓WC	[123]
Wild pistachio (*Pistacia atlantica*)	500 mg/day fruit powder; 2 months	T2DM patients, triple-blind	↓2hpp; ↓TG; ↓TC; ↓↓LDL-C; (NS) FBG, HbA1c; (NS) TG, HDL-C, ALT, AST, Cr	[124]
Lemon balm (*Melissa officinalis*)	1.4 g extract/day; 8 weeks	T2DM patients, DB	↑Apo A-I; ↓TC/HDL-C; ↓LDL-C/HDL-C	[125]
Garlic (*Allium sativum*)	13.5 mg EO/day; 3 and 6 months	T2DM patients with TC > 200 mg/dL	↓FBG; ↓HbA1c; ↓↓TC	[126]

2hpp, 2-h postprandial blood glucose; HbA1c, glycosylated hemoglobin; FBG, fasting blood glucose; BMI, body mass index; WC, waist circumference; BW, body weight; TG, plasma triglycerides; TC, total plasma cholesterol; LDL-C, low-density lipoprotein-cholesterol; HDL-C, high-density lipoprotein-cholesterol; ALT, alanine aminotransferase; AST, aspartate aminotransferase; GOT, glutamate transaminase; SBP and DPB, systolic and diastolic blood pressure; Cr, creatinine; Apo A-I, apolipoprotein A; NS, not statistically significant; DB, double-blind study; T2DM, type 2 diabetes mellitus; ↑ or ↓, variation within +/– 20% the initial value; ↑↑ or ↑↑, variation > 20%.

**Table 3:** Representative clinical trials of the impact of essential oils on mood and cognitive performance.

Essential Oil	Treatment	Study model	Effects	Ref.
Sage, 2 types (*S. officinalis* and *S. lavandulaefolia*)	5 drops of EO in 5 mL water/inhalation	DB on 45 healthy adult volunteer per EO or placebo/135 total	↑mood; ↑cognitive performance; ↑memory	[161]
Sage (*S. lavandulaefolia)*	25 μL or 50 μL EO/oral administration	Placebo-controlled, DB crossover on 24 volunteer students	↑mood ↑cognitive performance; ↓ catabolism of ACh	[165]
Rose (*R. damascena*) or orange (*C. sinensis*)	Exposure for 90 s to air impregnated with rose or orange	oxy-Hb using near-infrared TRS on 20 female university students	↓ oxy-Hb in the right prefrontal cortex; ↑subjective reports of relaxed feeling	[166]
Bitter orange (*C. aurantium)*	0.1 ml EO in 1.9 ml of distilled water by inhalation	Randomized on healthy subjects exposed to an anxiogenic task: crack users in abstinence	↓DBP; ↓HR; ↓ autonomic excitability; acute anxiolytic effects	[167]
Sweet orange *(C. aurantium)*	Inhalation for 15–20 min 1 h before intervention	SB randomized/80 patients undergoing coronary angiography	↓mean score of STAI; ↓SBP; ↓DBP; ↓respiratory and pulse rate	[168]
Lemon (*Citrus limon*)	Repeated inhalation: 4–6 sessions of 1 h/4 days	59 healthy students, DB, computer-based objective cognitive tests	↓reaction time; ↓memory sensibility; faster responses at the cost of accuracy	[169]
Bergamot (*Citrus bergamia*)	80 mg of EO trans-epidermally/4 weeks	Randomized, DB, 134 patients aged ≥ 65 y with severe dementia	Target: ↓agitation in severely demented elderly. The trial in ongoing.	[164]
Bergamot (*Citrus bergamia*)	Inhalation of 2%EO/twice a day	Placebo, single-blind test on 29 elementary school teachers	↓LF; ↓LF%;↓LF/HF	[170]
Chamomile + lavender (mixed)	Inhalation of mixed aroma diluted 5%	Randomized, DB on 120 nurses conducted between 2018 and 2019	↓anxiety; ↓depression; ↓stress (DASS scale)	[171]
Jasmine flower (*Jasminum spp.*)	Inhalation of EO/60 min before	Patients undergoing laparotomy; SB parallel, randomized	↓cortisol; ↓mean score anxiety	[172]
Peppermint (*Mentha piperita*)	Nasal drop application of EO 1.5% vs. lidocaine 4%	Randomized, DB, controlled crossover on 120 patients with migraine	↓headache frequency; ↓headache intensity; (similar to lidocaine)	[173]
Peppermint (*Mentha piperita*)	Inhalation of 3 drops of EO/7 nights	Randomized controlled on 105 cardiac patients	↓average fatigue, (not different from lavender)	[174]
Lavender (*L. angustifolia*)	Inhalation 3 drops of EO/7 nights	Randomized controlled on 105 cardiac patients	↓average fatigue, (not different from peppermint)	[174]
Spearmint (*Mentha spicata*)	Physical edu. students inhalation	Quasi-experimental, uncontrolled, before and after	↑forced expiration vol.; ↑lung status; ↑spirometry; ↓running time	[175]
Sweet orange (*Citrus sinensis*)	Physical edu. students inhalation	Quasi-experimental, uncontrolled, before and after	↑forced expiration vol.; ↑lung status; ↓running time	[175]
Lemon balm (*M. officinalis*)	Inhalation 2 drops of EO diluted in oil	DB controlled, 72 patients suffering from ACS	↓mean score of stress; ↓HR; ↓MAP	[176]
Lavender (*Lavandula spp.*)	Exposure to airborne organic EO	30 healthy students performing serial arithmetic task	↓salivary CgA no significant change in level of Cortisol	[177]
Lavender (*L. angustifolia*)	Inhalation for 5 min at bedtime/8 weeks	52 patients with T2DM and insomnia, randomized crossover	↑sleep quality and quantity, ↑quality of life; ↑mood. No significant effect on metabolic status	[178]
Lavender *(L. angustifolia*)	EO 1 drop on the pillow, twice a week for one month	49 obese women randomized, data collected in two moments on VAS for pain	↓total spinal pain; ↓cervical region pain; ↓lumbar region pain	[179]
Lavender (*Lavandula spp.*)	10 drops of EO in 1 L of water, for 15 min by humidifier	Single-blind randomized, 33 nursing students/anxiety (TAI)	No positive effects on students	[180]
Lavender, Roman chamomile, and neroli 6:2:0.5 ratio	Inhalation of 2 drops of EO blend, 10 times	56 patients in ICU nonequivalent control group, quasi-experimental, before and after	↓anxiety; ↑sleep quality; No significant difference on BP	[181]
Akita cedar (*C. japonica*)	Inhalation/8 weeks	36 AD patients, randomized	↑NPI score; ↑J-ZBI; (NS) ADAS-cog score	[182]

CgA, chromogranin A; LF, low frequency power; HF, high frequency power; ACS, acute coronary syndrome; oxy-Hb, oxygenated hemoglobin; HR, heart rate; MAP, mean arterial pressure; STAI, Spielberger’s State–Trait Anxiety Inventory; SBP, systolic blood pressure; DBP, diastolic blood pressure; T2DM, type 2 diabetes mellitus; TAI, test anxiety inventory; VAS, visual analog scale; ICU, intensive care unit; BP, blood pressure; NPI, neuropsychiatric inventory; J-ZBI, Zarit caregiver burden interview; AD, Alzheimer’s disease; NS, not significant; DB, double-blind; SB, single blind; ↑ or ↓, significant variation of initial condition.

## Supplementary Materials

The following supporting information can be downloaded at: https://www.mdpi.com/article/10.3390/molecules28020901/s1, Table S1: Examples of common Essential Oils (including different chemotypes, CT) used in food products.
