# Supplementary material for: Plant Essential Oils as Healthy Functional Ingredients of Nutraceuticals and Diet Supplements: A Review
Source: Molecules. 2023 Jan 16;28(2):901. doi: 10.3390/molecules28020901 (PMC9862182; doi:10.3390/molecules28020901)
Supplement: Supplementary file 1 [file molecules-28-00901-s001.zip › molecules-2125147-supplementary.pdf]

Plant Essential Oils as Healthy Functional Ingredients of Nutraceuticals and Diet Supplements. A Review

Riccardo Matera [1], Elena Lucchi [1] and Luca Valgimigli [2,*]

[1] BeC s.r.l., Research & Development, Via C. Monteverdi 49, 47122 Forlì, Italy
[2] University of Bologna, Department of Chemistry "Ciamician" Via S. Giacomo 11, 40126 Bologna, Italy
* Correspondence: luca.valgimigli@unibo.it; Tel.: +30 051 2095683

Supplementary Materials

Table S1. Examples of common Essential Oils (including different chemotypes, CT) used in food products.

Plant EO	Parts used (extraction method)	Main and characteristic components (%)	Location	Ref.
Anise (*Pimpinella anisum*)	Fruits (steam distillation)	*t*-Anethole (78-94%), γ-himachalene (0.4–8.2%), p-anisaldehyde (tr-5.4%), methylchavicol (0.5–2.3%).	Italy, France, Greece, Hungary	[38]
Basil (*Ocimum basilicum)* CT: linalool/eugenol	Leaves (steam distillation)	Linalool (35-66%), eugenol (8-30%); others: 1,8-cineole (4-10%), α-*t*-bergamotene (1-5%), *t*-ocimene (0.5-3%), cadinol (1-6%)	Italy, Croatia, Albania	[187]
Basil (*Ocimum basilicum)* CT: citral/linalool	Leaves (steam distillation)	Citral (47-72%), linalool (6-11%); others: 1,8-cineole (0.2-1%), eugenol (0.1-0.5%), estragol (tr-5%), nerol (tr-5%), *t*-ocimene (tr-0.3%)	Italy, Romania	[187]
Basil (*Ocimum basilicum)* CT: linalool/1,8-cineole	Leaves (steam distillation)	Linalool (30-73%), 1,8-cineole (5-23%); others: α-terpineol (0.1-1%), cis-thujone (2-7%), eugenol (1-3%), epi-α-cadinol (3-4%), α-*t*-bergamotene (4-10%)	Brasil, Greece, India, Egypt, Mississipi	[187]
Bergamot (*Citrus bergamia*)	Fruit outer peel (cold pressing)	Limonene (30-50%), linalyl acetate (23-35%), β-pinene (5-10%), α-pinene (0.7-2%), γ-terpinene (6-18%), linalool (6-15%)	Italy	[164]
Chamomile, Roman (*Antemis nobilis*) CT: isobutyl angelate	Flowers (steam distillation)	Isobutyl angelate (25-35%), isoamyl angelate (1-23%), 2-methyl butyl angelate (5-18%), isobutyl butyrate (0.1-2%)	France	[188]
Chamomile, German (*Matricaria chamomilla*) CT: α-bisabolol	Flowers (steam distillation)	α-Bisabolol (30-60%), α-bisabolol oxide A (2-5%), *t*-*t*-farnesol (10-16%), cis-β-farnesene (5-7%), chamazulene (0.5-4%)	Europe, Iran	[188]
Cumin, Green (*Cuminum cyminum*)	Fruits (steam distillation)	Cuminaldehyde (42%), p-cymene (16%), γ-terpinene (17%), β-pinene (11%), 2-caren-10-al (3%), cis-dihydrocarvone (3%)	Iran	[114]
Cumin, Black (*Nigella sativa*)	Seeds (steam distillation)	p-Cymene (19-48%), thymol (13-45%), thymoquinone (3-5%), β-elemene (1-11%), thujene (tr-11%)	Iran	[121–123]
Coriander, Fruit (*Coriandrum sativum*)	Mature Fruits (steam distillation)	Linalool (65-79%), γ-terpinene (4-7%), camphor (3%), geranyl acetate (2-4%), geraniol (1-3%), α-pinene (1-3%), limonene (1-2%)	Italy	[57]
Coriander, Leaves (*Coriandrum sativum*)	Leaves (steam distillation)	2-(*E*)-decenal (12-16%), decanal (9-12%), 2-(*E*)-dodecenal (6-16%),	Kenya, Massachusetts	[57]

		2-(*E*)-dedecen-1-ol (8-14%), undecanal (2-3%), dodecanal (4-5%)		
Garlic (*Allium sativum*)	Bulb (steam distillation)	Diallyl disulfide (41%), diallyl trisulfide (22%), diallyl sulfide (7%), others: (allyl methyl trisulfide, vinyldithiins, ajoene)	Spain	[129]
Lavender (*L. angustifolia*) ISO 3515:2002	Flowers, leaves or aerial parts (steam distillation)	Linalool (25-38%), linalyl acetate (25-45%), 1,8-cineole (1-2%), camphor (0.5-1%), β-ocimene (2.5-6%), terpinen-4-ol (2-6%), lavandulyl acetate (3.4-6.2%)	Italy, France, England, Bulgary, Greece	ISO
Lavender (*L. angustifolia*) Pharmaceutical	Flowers (steam distillation)	Linalool (20-45%), linalyl acetate (25-46%), 1,8-cineole (<2.5%), camphor (<1.2%), terpinen-4-ol (0.1-0.6%), limonene (<1%) lavandulyl acetate (>0.2%)	Europe, USA	Eur. Ph.
Lemon (*C. limon*)	Fruit outer peel (cold pressing)	Limonene (65-85%), sabiene (5-15%), γ-terpinene (3-12%), α-pinene (1-3%) β-pinene (1-3%), neral (0.2-1%), geranial (0.2-1%), β-caryophyllene (0.1-1%)	Italy	[5]
Lemon balm (*Melissa officinalis*) CT: citral	Leaf, top (steam distillation)	Geranial (6-51%), neral (4-35%), geranyl acetate (0.5-19%), citronellal (0.4-20%), β-caryophyllene (1-7%), caryophyllene oxide (1-44%), α-cadinol (5-6%), α-copaene (0.1-7%), germacrene D (0.2-2%)	Iran, Tunisia, Germany, France	[128]
Myrtle (*Myrtus communis*)	Leaves (hydrodistillation)	Myrtenyl acetate (31%), linalool (15%), α-pinene (11%) and 1,8-cineole (10%), linalyl acetate (5%)	Tunisia	[95]
Neroly (*Citrus aurantium* var. *amara*) ISO 3517:2002	Flowers (steam distillation)	Linalool (28-44%), limonene (8-18%), myrcene (1-4%), (*E*)-β-ocimene (3-8%), β-pinene (7-17%), α-terpineol (2-6%), farnesol (1-4%), nerolidol (1-5%), geranyl acetate (1-5%), neryl acetate (tr-2.5%), nerol (1%), geraniol (3%)	Tunisia, Morocco, Egypt, Italy	ISO
Orange, Bitter (*C. aurantium* var. *amara*)	Fruit outer peel (cold pressing)	Limonene (94%), β-myrcene (2.4%), α-pinene (0.6%), β-pinene (0.7%), linalyl acetate (0.8%), γ-terpinene (0.3%)	Italy	[49]
Orange, Sweet (*C. sinensis* or *C. aurantium* var *dulcis*)	Fruit outer peel (cold pressing)	Limonene (78%), β-myrcene (3.1%), linalool (1.7%), α-pinene (1.4%), sabinene (1%), decanal (1%), neryl acetate (0.5%), geranial (0.4%), carvone (0.8%)	Brasil	[166]
Rosemary (*R. officinalis*) CT: cineole/camphor/α-pinene	Leaves (hydrodistillation)	1,8-Cineole (44%), camphor (13%), α-pinene (12%), β -pinene (8%), camphene (5%), β-caryophyllene (4%), borneol (3%)	Serbia	[132]
Peppermint (*Mentha piperita*)	Leaves (steam distillation)	Menthol (50-60%), menthone (14-32%), menthyl acetate (2.8-10%), isomenthone (1.5-10%), menthofuran (1-9%), 1,8-cineole (3.5-14%), limonene (1-5%)	Europe, America	[33]
Pine (*Pinus sylvestris*)	Wood and leaves (steam distillation)	α-Pinene (20-30%), δ-3-carene (10-24%), β-pinene (1-5%), camphene (2-5%), terpinolene (1-2%), epi-cubebol (3-5%), β-caryophyllene (3-5%), germacrene D(2-7%)	Europe, USA	[5]
Sage (*Salvia officinalis*)	Leaves (steam distillation)	α-Thujone (17-27%), camphor (13-21%), 1,8-cineole (12-27%), β-thujone (4-6%),	Europe, America	[189]

CT: α-thujone/<br>camphor/1,8-cineole		β-caryophyllene (3-5%), α-Humulene (3-6%), viridiflorol (2-7%), limonene (1-2%)		
Thyme<br>(*Thymus vulgaris*)<br>CT: p-cymene/carvacrol	Leaf, top<br>(steam distillation)	p-Cymene (45%), carvacrol (34%), camphene (9%), thymol (4%), α-pinene (4%), β-pinene (3%)	Italy	[20]
Thyme<br>(*Thymus vulgaris*)<br>CT: thymol	Leaf, top<br>(steam distillation)	Thymol (37-67%), p-cymene (13-19%), γ-terpinene (6-19%), carvacrol (4-6%), myrcene (0.7-2.2%), linalool (1.8-2.2%)	Austria	[186]
Thyme<br>(*Thymus vulgaris*)<br>CT: thymol/carvacrol	Leaf, top<br>(steam distillation)	Thymol (23%), carvacrol (26%), p-cymene (11%), γ-terpinene (19%), myrcene (1.9%), linalool (6%), β-caryophyllene (1.5%)	Austria	[186]
Yarrow<br>(*Achillea millefolium*)<br>CT: Germacrene D<br>/sabinene/β-pinene	Flowers<br>(steam distillation)	Germacrene D (26%), sabinene (14), β-pinene (11%), β-caryophyllene (10%), chamazulene (10%), borneol (5%),	Iran	[85]
